# Novel Coumarin–Pyridine Hybrids as Potent Multi-Target Directed Ligands Aiming at Symptoms of Alzheimer’s Disease

**DOI:** 10.3389/fchem.2022.895483

**Published:** 2022-06-30

**Authors:** Elaheh Babaei, Tuba Tüylü Küçükkılınç, Leili Jalili-Baleh, Hamid Nadri, Esin Öz, Hamid Forootanfar, Elaheh Hosseinzadeh, Tayebeh Akbari, Mehdi Shafiee Ardestani, Loghman Firoozpour, Alireza Foroumadi, Mohammad Sharifzadeh, Bi Bi Fatemeh Mirjalili, Mehdi Khoobi

**Affiliations:** ^1^ Department of Chemistry, Faculty of Science, Yazd University, Yazd, Iran; ^2^ Department of Biochemistry, Faculty of Pharmacy, Hacettepe University, Ankara, Turkey; ^3^ Department of Medicinal Chemistry, Faculty of Pharmacy and Pharmaceutical Sciences, Tehran University of Medical Science, Tehran, Iran; ^4^ Faculty of Pharmacy, Shahid Sadoughi University of Medical Sciences, Yazd, Iran; ^5^ Department of Pharmaceutical Biotechnology, Faculty of Pharmacy, Kerman University of Medical Sciences, Kerman, Iran; ^6^ The Institute of Pharmaceutical Sciences (TIPS), Tehran University of Medical Sciences, Tehran, Iran; ^7^ Department of Microbiology, Islamic Azad University, North Tehran Branch, Tehran, Iran; ^8^ Department of Radiopharmacy, Faculty of Pharmacy, Tehran University of Medical Sciences, Tehran, Iran; ^9^ Department of Toxicology and Pharmacology, Faculty of Pharmacy, Tehran University of Medical Sciences, Tehran, Iran

**Keywords:** Alzheimer’s disease, cholinesterase inhibitors, coumarin derivatives, neurodegenerative diseases, docking study

## Abstract

In this research, a series of coumarin-based scaffolds linked to pyridine derivatives *via* a flexible aliphatic linkage were synthesized and assessed as multifunctional anti-AD agents. All the compounds showed acceptable acetylcholinesterase (AChE) inhibition activity in the nanomolar range (IC_50_ = 2–144 nM) and remarkable butyrylcholinesterase (BuChE) inhibition property (IC_50_ = 9–123 nM) compared to donepezil as the standard drug (IC_50_ = 14 and 275 nM, respectively). Compound **3f** as the best AChE inhibitor (IC_50_ = 2 nM) showed acceptable BuChE inhibition activity (IC_50_ = 24 nM), 100 times more active than the standard drug. Compound **3f** could also significantly protect PC12 and SH-SY5Y cells against H_2_O_2_-induced cell death and amyloid toxicity, respectively, superior to the standard drugs. It could interestingly reduce β-amyloid self and AChE-induced aggregation, more potent than the standard drug. All the results suggest that compound **3f** could be considered as a promising multi-target-directed ligand (MTDL) against AD.

## Graphical Abstract

A novel series of 3-phenyl-coumarin derivatives containing pyridinium salts was prepared, indicating significant inhibitory activity against AChE and BuChE as well as precious neuroprotective and anti-Aβ aggregation effects.
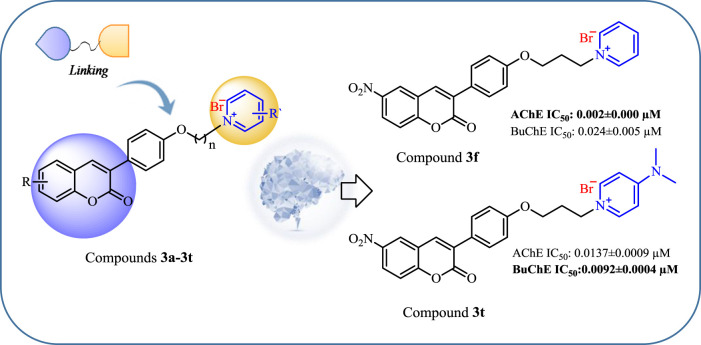

## Highlights

• Twenty novel coumarin–pyridine hybrids were synthesized as anti-Alzheimer agents
• All the synthesized compounds were assessed for their anti-cholinesterase activity, and all of them indicated acceptable inhibitory activity
• Compound **3f** was the most influential compound against AChE
• Compound **3f** was more active than standard drugs in the protection of PC12 and SH-SY5Y cells against H_2_O_2_-induced cell death and amyloid toxicity, respectively
• Compound **3f** was superior to the standard drug in the reduction of ß-amyloid self and AChE-induced aggregation

## 1 Introduction

Alzheimer’s disease (AD) is a destructive neurodegenerative irregularity affecting memory, way of thinking, speaking, and other behavioral activities (Wang et al., 2014). Today, millions of people are afflicted with this disease, and the number is projected to increase in the next few years (Sang et al., 2018). Different factors such as inflammation, reduced acetylcholine (ACh) concentration, β-amyloid (Aβ) plaque formation, τ-protein aggregation, and oxidative stress cause AD, while the main reason is not perfectly understood (Patel et al., 2020). One of the most significant factors is the declined cholinergic activity resulting from ACh degradation by acetylcholinesterase (AChE), which affects memory loss. Conventionally approved drugs are mostly AChE inhibitors, increasing the amount of ACh in the synapses and decelerating the advancement of AD (Reddy et al., 2017; Li et al., 2018; Patel et al., 2020). Moreover, studies on the structure of AChE have indicated that it has two main sites for binding, including a peripheral anionic site (PAS) and a catalytic anionic site (CAS). AChE inhibitors binding to these two sites are more influential than the inhibitors occupying just one site of the enzyme. Butyrylcholinesterase (BuChE) has also had a great effect on the advancement of AD. BuChE inhibitors could retrieve cholinergic activity via restoring the proportions of AChE/BuChE activity like in a normal brain (Liu et al., 2020). As a consequence, dual AChE/BuChE inhibitors have drawn great interest in the management of AD (Davidsson et al., 2001).

On the other hand, AD is a continuing dementia equated by selective neuronal cell demise, which is undoubtedly triggered by Aβ fibrils or oligomers (Selkoe and Hardy, 2016; Murakami et al., 2020; Trambauer et al., 2020). Furthermore, several biomedical studies have shown that AChE promotes formation of amyloid fibril and creates extremely poisonous AChE-Aβ complex throughout the peripheral anionic site (PAS) (Inestrosa et al., 2005; Pradhan et al., 2018). Controlling Aβ protein formation or aggregation has a significant role in the enhancement of AD (Zou et al., 2015; Vyas et al., 2018). In primary steps of the degenerative nerve process, Aβ could go into the mitochondrion and escalate the creation of reactive oxygen species (ROS) by interrupting the electron transport chain, provoking oxidative stress (Chen et al., 2016; Yang et al., 2020). Hence, inhibition of Aβ accumulation, and therefore the formation of free radicals or preserving the cells against oxidative stress by neuroprotective agents, will be a propitious approach for the management of AD.

Advancement of multi-target-directed ligands (MTDLs) is one of the most encouraging drug discovery methods for ailments with a complex nature like AD. Mono-targeted medicines could not forever amend the complex ailing system sufficiently, even if these compounds regulate their targets with extreme selectivity and affinity (Zhang et al., 2016; Alcaro et al., 2019; González et al., 2019; Li et al., 2020). MTDLs have a superior capability to exert influence on the complicated balance of entire cellular network than single-targeted drug due to their synchronous consequences on various curative targets. Another positive aspect of these beneficial drugs is that they have a greater efficiency/security ration than a one-targeted drug (Bolognesi et al., 2011; Xuan et al., 2021). Consequently, there is a necessity to design suchlike compounds that can be effective on diverse relevant targets of AD, concurrently. These kinds of properties can be presumably attained by the linkage of diverse active moieties impacting on several targets. The linking hybrids with impression on distinctive targets might be advantageous to treat a complicated disorder like AD (Cavalli et al., 2008; Du et al., 2019; Sharma et al., 2019). Hitherto, the only three available ChE inhibitors prescribed for AD management (donepezil, rivastigmine, and galantamine) are single-target compounds inhibiting only ChEs. But they have no appropriate efficacy and may not be clinically significant (Marucci et al., 2021). In addition, so-called “drug-cocktails” like Memantine plus cholinesterase inhibitors are used in patients with advanced disease to pharmacologically treat these pathologies, with concerns related to drug-drug interactions as well as patient compliance (Chen et al., 2017). Hence, according to the paradigm of “network pharmacology,” further research is needed to find novel bioactive compounds with multi-target properties (Hopkins, 2008; Chen et al., 2017). Irrevocably, treating AD and other neurodegenerative disease has been one of the major focal points of multi-target drug discovery procedures in the past 20 years.

A wide range of coumarin derivatives has been associated with an antioxidant trait (Bilgin et al., 2011; Anand et al., 2012; Pérez-Cruz et al., 2018; Koyiparambath et al., 2021). Some of which are identified to be active as AChE inhibitors and, as a consequence, could be noticed as a candidate with potential for the management of AD (Canning et al., 2013; Nasr et al., 2014; Bagheri et al., 2015). Additionally, a coumarin-based molecule, ensaculin, which contains benzopyran with the substitution of a piperazine, was introduced with the ability to enhance cognition and memory functions (Witaicenis et al., 2014; Abu-Aisheh et al., 2019).

On the other hand, investigations have been focused on the pyridine moiety because of its biological properties such as antioxidant and anti-inflammatory activities (Altaf et al., 2015). Furthermore, pyridinium salt has a well-known role in pharmacological interaction such as potent binding attraction in the direction of catalytic active site (CAS) of AChE by usage of charge interactions, π-stacking and therefore diverse MTDLs creating suchlike moiety have been reported so far (Kapková et al., 2006; Vafadarnejad et al., 2018; Mollazadeh et al., 2019).

**FIGURE 1:**
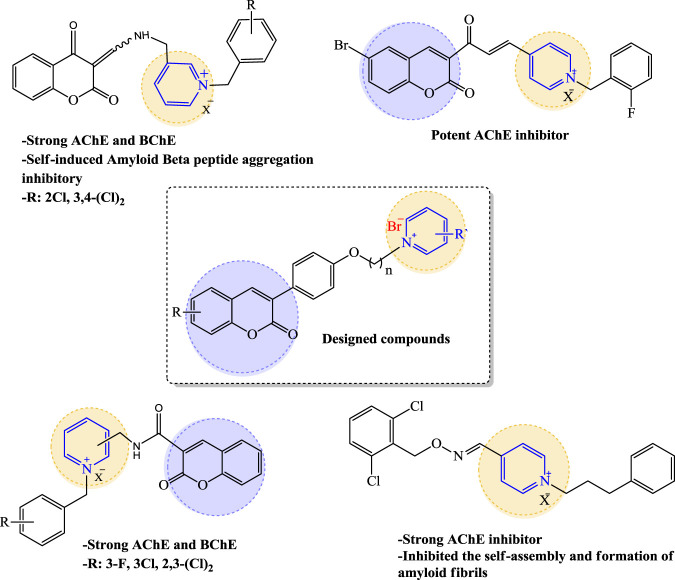
Design strategy for the preparation of compounds **3a–3t**.

In this work and in progression of our interest in the development of potent multi-target derivatives against AD (Choubdar et al., 2019; Abdpour et al., 2021), novel coumarin derivatives cross-linked with pyridinum salts were designed and synthesized by a simple procedure, resulting in a high yield. We focused on improving ChE inhibition of the coumarin-pyridinium backbone via a range of derivatives with donor/acceptor properties of the substituents, along with a flexible carbon chain as a linker to dedicate the structure of the target molecules with proper binding attraction and flexibility towards ChE enzymes as well as anti-Aβ aggregation and neuroprotective activities (Figure 1). We hope we can pave the way for rational and potent multi-target small molecule discovery in AD management, free from concerns about the cost, safety, and tolerability profile of the peptides and antibodies (Jeremic et al., 2021).

## 2 Results and Discussion

### 2.1 Chemistry

**SCHEME 1:**
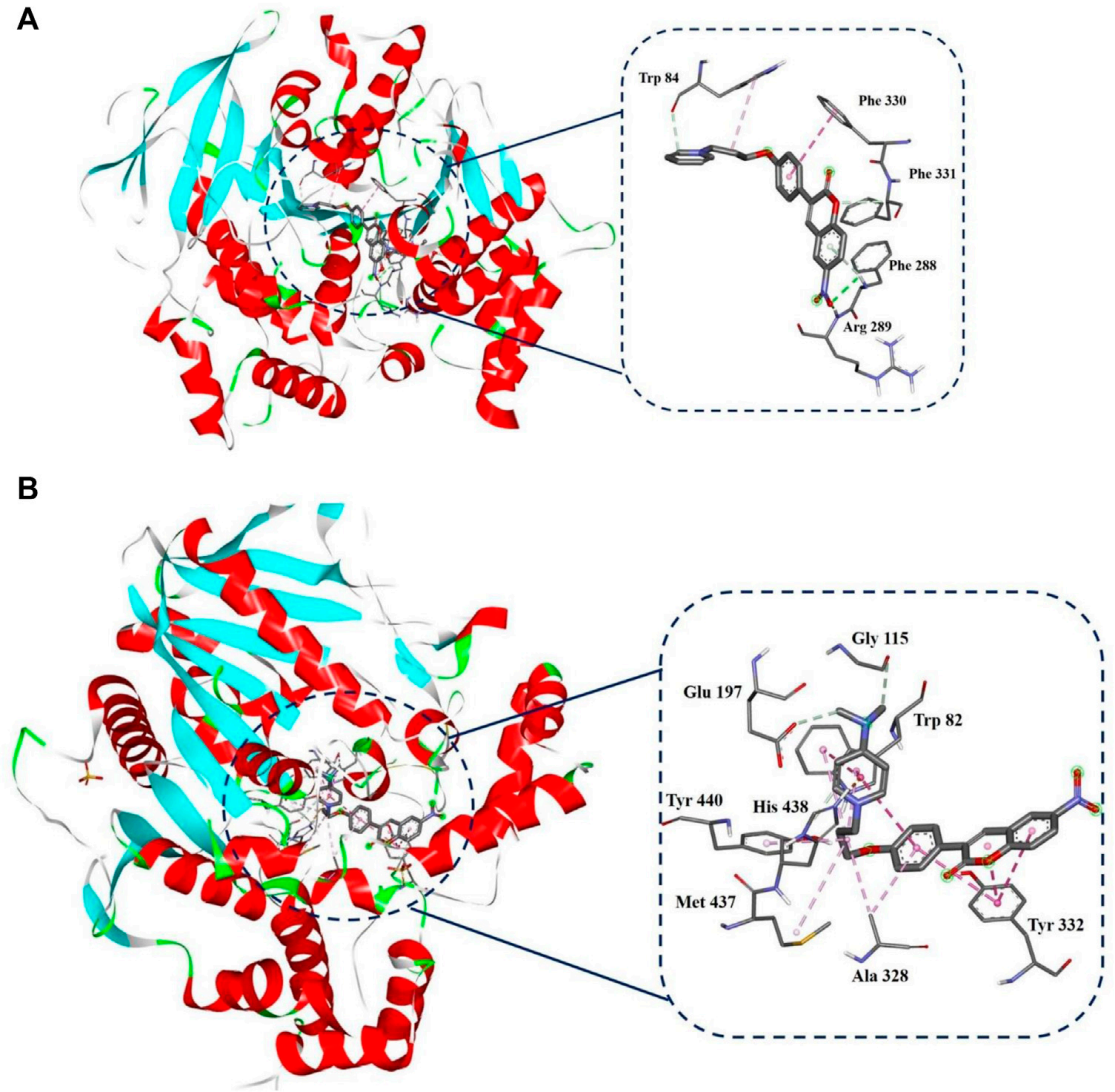
Molecular docking of AChE **(A)** and BuChE **(B)** binding with compounds 3f and 3t, respectively.

All products were synthesized using commercially available 4-Hydroxyphenylacetic acid. Compound **1** was initially synthesized *via* the reaction of 4-hydroxyphenylacetic acid with different salicylaldehyde derivatives in the presence of sodium acetate (NaOAc) in acetic anhydride (Ac_2_O) under reflux conditions. The intermediate **1** was then reacted with an excess amount of various alkyl dibromides, potassium carbonate (K_2_CO_3_) in dry acetone under reflux condition, followed by reaction with different substituted pyridine in neat condition at 80°C to obtain the desired target compounds **3a**-**q** (Scheme 1). Also, products **3r-t** were directly synthesized from the reaction of compound **2** with 4-dimethylaminopyridine in dry acetonitrile under reflux conditions. All the reactions were monitored by TLC, and the products were purified simply by adding ether to the reaction solution. IR, ^1^H and ^13^C NMR spectroscopy were utilized for the product characterization.

### 2.2 Biological Assays

#### 2.2.1 Anti-Cholinesterase Activity

To evaluate the anti-AD activity of the target compounds, their abilities to inhibit AChE and BChE were evaluated. All derivatives were screened for their *in vitro* AChE and BChE inhibitory properties (Table 1). The results showed that all the tested compounds were much more effective than Donepezil as the standard drug, with excellent AChE inhibitory activity in the nano-molar range. In particular, compounds **3p**, **3l**, **3a**, **3b**, **3c**, and **3g** showed inhibitory activities with IC_50_ values of 3, 3, 4, 4, 7, and 7 nM respectively. Compound **3f,** as the best AChE inhibitor with an IC_50_ value of 2 nM, was 7 times more active than the standard drug, Donepezil. The results revealed that 3-carbon chain length (*n* = 3) could be considered as the optimum length of the linker for AChE inhibition and that elongation of the linker led to a decrease in the activity. Also, the presence of different substituents at the phenyl ring of the coumarin moiety (R) had no effective role in improvement of the inhibitory effect, except for the nitro group on the 6-position of the coumarin ring in compounds **3f** and **3l,** which were the most potent compounds. However, increasing the length of the linker (*n* = 5, compound **3o**) and the presence of a 4-dimethylamine group at the para position of the pyridinium ring (compound **3t**), reduced the AChE inhibitory effect of the compounds having a 6-NO_2_ group. We also investigated the BuChE inhibitory activity of the isolated compounds (Table 1). Most of the compounds had high BuChE inhibition activity in the range of 22–123 nano-molar. But compound **3t** exhibited remarkable BuChE inhibitory activity with an IC_50_ = 9 nM, 305 times more active than the standard drug, Donepezil (BuChE IC_50_ = 2,750 nM). Compounds **3a-b, 3f-g, 3l, and 3t** as the best AChE and BuChE inhibitors were selected for further analysis.

**TABLE 1:** Inhibitory activity of the target compounds **3a-t** against AChE and BuChE.

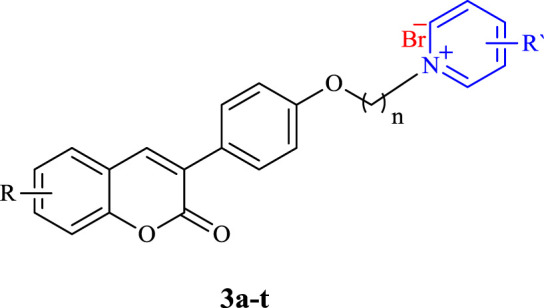					
**Compound**	**n^c^**	**R**	**R′**	**AChE IC_50_ (nM)^a^**	**BuChE IC_50_ (nM)^b^**
**3a**	3	H	H	4.0 ± 0.0	84.0 ± 4.0
**3b**	3	6-OMe	H	4.0 ± 1.0	68.0 ± 6.0
**3c**	3	7-OMe	H	7.0 ± 1.0	57.0 ± 10.0
**3d**	3	8-OMe	H	81.0 ± 4.0	79.0 ± 9.0
**3e**	3	6-Br	H	15.0 ± 3.0	33.0 ± 5.0
**3f**	3	6-NO_2_	H	2.0 ± 0.0	24.0 ± 5.0
**3g**	4	H	H	7.0 ± 1.0	91.0 ± 18.0
**3h**	4	6-OMe	H	1003 ± 37.0	106.0 ± 8.0
**3i**	4	7-OMe	H	144.0 ± 9.0	46.0 ± 1.0
**3j**	4	8-OMe	H	31.0 ± 5.0	97.0 ± 19.0
**3k**	4	6-Br	H	40.0 ± 5.0	92.0 ± 9.0
**3l**	4	6-NO_2_	H	3.0 ± 1.0	59.0 ± 11.0
**3m**	5	H	H	72.0 ± 8.0	86.0 ± 10.0
**3n**	5	8-OMe	H	15.0 ± 2.0	123.0 ± 24.0
**3o**	5	6-NO_2_	H	22.0 ± 4.0	27.0 ± 2.0
**3p**	5	6-Br	H	3.0 ± 0.0	49.0 ± 5.0
**3q**	5	6-Br	5-Ethyl-2-Methyl	19.0 ± 1.6	22.0 ± 2.3
**3r**	5	6-Br	4-Dimethylamine	26.0 ± 2.1	52.0 ± 3.6
**3s**	3	H	4-Dimethylamine	22.0 ± 1.9	34.0 ± 2.6
**3t**	3	6-NO_2_	4-Dimethylamine	14.0 ± 0.9	9.0 ± 0.4
**Donepezil**				14.0 ± 3.0	2,750 ± 205.0

a Inhibitor concentration (mean ± SEM of three experiments) required for 50% inactivation of AChE (electric eel).

b Inhibitor concentration (mean ± SEM of three experiments) required for 50% inactivation of BuChE (equine serum).

c Length of linker (*n* = 3, 4, 5).

#### 2.2.2 Ligand–Protein Docking Simulation

**FIGURE 2:**
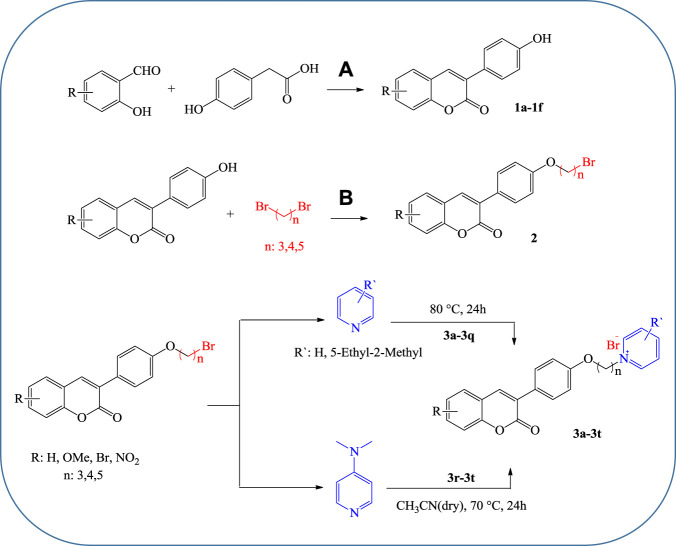
Synthesis of target compounds 3a–t. **(A)** Ac_2_O/NaOAc/Reflux 4–6 h, **(B)** K_2_CO_3_/acetone (dry)/Reflux 4 h

To have a deep understanding of the interactions of potent compounds (**3f** and **3t**) with AChE and BuChE targets, respectively, docking simulation was carried out (Figure 2). In the case of AChE, compound **3f** showed interactions with Trp84 at the anionic site, hydrophobic Pi-Pi stacking interactions with Phe330 and two hydrogen bonds with Phe288 and Arg289 with the nitro group (Figure 2A). Therefore, compound **3f** inhibited AChE through hydrophobic interactions, and it was stabilized in the active site through hydrogen bonds.

Regarding the most active BuChE inhibitor, compound **3t** showed Pi-Pi stacking interactions with Tyr332 and Trp82 residues, Pi-cation interactions with His438 in the vicinity of the pyridinium region with positive charge, and Van der Waals interactions with Phe329, Pro285, Ser79, Gly78, Trp430, Gly439, Tyr128, Gly116, and Asp70 residues (Figure 2B). Therefore, it seems that hydrophobic interactions are responsible for the interactions of BuChE with compound **3t**.

#### 2.2.3 Neuroprotection Assay Against H_2_O_2_-Induced Cell Death in PC12 Cells

The neuroprotective effects of compounds **3a-b, 3f-g, 3l, and 3t** at the concentrations of 0.1, 1, 5, 10, 20, and 50 μM against neurotoxicity caused by H_2_O_2_ were evaluated on neuroblastic PC12 cells. All the tested concentrations increased PC12 cell viability in a concentration-dependent manner (Table 2). Compounds **3a**, **3f**, **3l,** and **3t** showed a greater effect on cell viability, especially at low concentrations. Notably, the neuroprotective effects of compounds **3f** and **3l** at all concentrations were higher than those of the reference drug, Quercetin.

**TABLE 2:** The protective effect of compounds 3a, 3b, 3f, 3g, 3l, and 3t against H_2_O_2_ (150 μM)-induced injury in the PC12 cell line at different concentrations in comparison to Quercetin^a^.

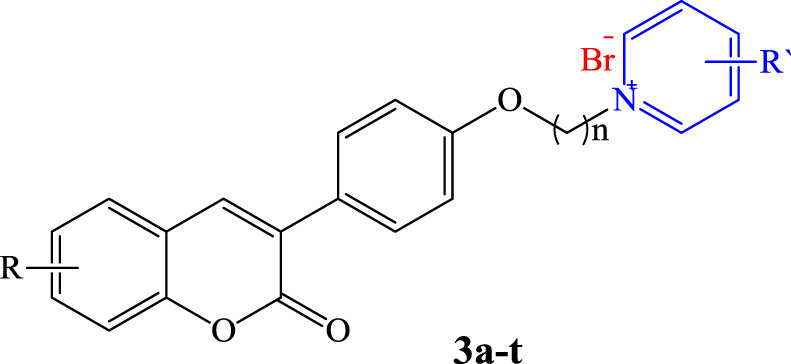										
**Compound**	**n**	**R**	**R′**	**PC12 cell viability (% of control)**						
				**H**_**2**_ **O**_**2**_	**0.1 μM**	**1 μM**	**5 μM**	**10 μM**	**20 μM**	**50 μM**
**3a**	3	H	H	26.7 ± 0.8	39.4 ± 1.7	42.2 ± 1.3	53.6 ± 1.4	55.6 ± 0.1	57.4 ± 0.4	58.3 ± 1.4
**3b**	3	6-OMe	H	26.2 ± 0.2	31.7 ± 1.4	37.6 ± 1.1	43.3 ± 0.9	45.4 ± 1.3	49.6 ± 0.9	54.3 ± 1.6
**3f**	3	6-NO_2_	H	25.4 ± 1.5	34.1 ± 1.0	42.8 ± 1.4	43.1 ± 1.5	45.0 ± 0.8	48.3 ± 0.4	50.8 ± 0.6
**3g**	4	H	H	25.5 ± 0.7	30.2 ± 1.4	33.6 ± 1.2	36.6 ± 0.9	45.8 ± 0.3	48.7 ± 0.9	49.9 ± 0.9
**3l**	4	6-NO_2_	H	23.5 ± 1.0	23.7 ± 0.5	27.3 ± 1.2	33.5 ± 1.2	37.8 ± 1.0	51.6 ± 1.1	55.7 ± 2.0
**3t**	3	6-NO_2_	4-Dimethylamine	22.9 ± 0.7	38.3 ± 0.9	45.3 ± 0.7	53.6 ± 0.7	56.3 ± 1.6	68.2 ± 0.1	71.8 ± 1.1
**Quercetin**	—	—	—	28.0 ± 0.8	37.7 ± 1.2	44.2 ± 0.7	50.7 ± 0.1	55.8 ± 0.3	59.6 ± 0.8	61.0 ± 0.8

a Cell viability was determined using the MTT assay protocol. Data are expressed as the mean ± SEM of three independent replicates.

#### 2.2.4 Cytotoxicity and Neuroprotection Against Aβ_1–42_-Induced Cytotoxicity in SH-SY5Y Cells

The cytotoxic effects of the best neuroprotective compounds **3a**, **3f,** and **3t** at a concentration of 1 μM were also evaluated on SH-SY5Y cells (Table 3). The results showed that all three compounds showed no cytotoxic effect on SH-SY5Y cells. Additionally, the potential neuroprotective effect of compounds **3a**, **3f,** and **3t** against Aβ_1-42_-induced cytotoxicity in SH-SY5Y cells was evaluated at a concentration of 1 μM. Compounds **3a** and **3f** could increase cell viability in the presence of Aβ and protect neuronal cells against Aβ toxic effects (Table 4). However, compound **3t** could not protect neuronal cells at a concentration of 1 μM. According to the results, compounds **3a** and **3f** showed protective capability with acceptable cell viability of 79% and 89%, respectively, at concentrations of 1 μM, higher than donepezil as the reference drug (77%).

**TABLE 3:** The cytotoxic effect of selected compounds **3a**, **3f,** and **3t** on the SH-SY5Y cell line.

Compound	n	R	R′	SH-SY5Y cell viability (% of control)^a^
				1 µM
**3a**	3	H	H	81.2 ± 10.0
**3f**	3	6-NO_2_	H	83.6 ± 3.4
**3t**	3	6-NO_2_	H	84.8 ± 6.8

a Cell viability is expressed as the mean percentage of viable cells compared with the untreated cells using the MTT assay protocol. The data are the mean ± SEM.

**TABLE 4:** The protective effect of compounds **3a**, **3f,** and **3t** against amyloid-induced injury in the SH-SY5Y cell line in comparison to Donepezil^a^.

Compound	n	R	R′	SH-SY5Y cell viability (% of control)
				1 µM
**3a**	3	H	H	79.1 ± 4.0
**3f**	3	6-NO_2_	H	89.0 ± 9.8
**3t**	3	6-NO_2_	H	59.7 ± 2.8
**Donepezil**	—	—	—	77.1 ± 3.6
**Aβ**_**1-42**_	—	—	—	72.2 ± 7.5

a Protective effects of compounds **3a**, **3f** and **3t** on cell injury induced by Aβ_1-42_ in SH-SY5Ycells. All groups were treated with 5 μM Aβ_1-42_ except for the control group. The synthetic compounds and Donepezil were pre-incubated at 1 µm of concentration in serum-free media for 24 h before the addition of Aβ peptide. Cell viability is expressed as the mean percentage of viable cells compared with the untreated cells. The data are the mean ± SEM.

#### 2.2.5 Inhibitory Potency of the Compounds Against Self-Induced and AChE-Induced Aβ_1-42_ Aggregation

The potential of compounds **3a**, **3f,** and **3t**, as the most active compounds based on the performed analyses, to inhibit Aβ-aggregation was evaluated using the thioflavin T (ThT) assay. The results indicated that the tested compounds displayed inhibitory activity about 2-fold more effective than the reference drug donepezil against Aβ aggregation (30.8% inhibition for donepezil vs. 73.3%, 84.7%, and 66.4% inhibition for compounds **3a**, **3f,** and **3t**, respectively, Table 5). The potential of compounds **3a**, **3f,** and **3t** to inhibit Aβ aggregation induced by AChE was also evaluated (Table 5). Compounds **3a**, **3f,** showed good inhibition activity toward AChE-induced Aβ aggregation (76.0% and 87.2% inhibition, respectively) more than donepezil as the standard drug (71.9% inhibition), except for compound **3t,** which was less active (52.5% inhibition) than donepezil. Compound **3f,** as the most active BuChE inhibitor, exhibited the most inhibition activity against both self and AChE-indued Aβ aggregation (84.7% and 87.2% inhibition, respectively).

**TABLE 5:** Inhibition of Aβ self- and AChE-induced aggregation by the compounds **3a**, **3f,** and **3t**.

Compound	Inhibition of self-induced Aβ aggregation^a^(%)	Inhibition of AChE-induced Aβ aggregation^b^(%)
**3a**	73.3 ± 15.4	76.0 ± 1.6
**3f**	84.7 ± 1.6	87.2 ± 5.7
**3t**	66.4 ± 4.7	52.5 ± 4.9
**Donepezil**	30.8 ± 1.7	71.9 ± 1.2

a Inhibition of self-induced Aβ_1-42_ aggregation (20 μM) produced by the tested compound at 100 μM concentration after 48 h. Values are expressed as means ± SEM of three experiments.

b Co-aggregation inhibition of Aβ_1-42_ and AChE (0.01 u/ml) by the tested compound at 100 μM concentration was detected by ThT assay. Values are expressed as means ± SEM of three experiments.

## 3 Conclusion

In this work, coumarin derivatives were cross-linked to pyridinium salts via flexible aliphatic carbon chains, and the target compounds were evaluated as MTDLs against AD. All the compounds showed high AChE and BuChE inhibition activity in the nano-molar range. Compound **3f** exhibited 7 times more AChE inhibition activity and compound **3t** had a 305 times greater inhibitory effect against BuChE compared to the standard drug. Especially, compound **3f** as the best AChE inhibitor (IC_50_ = 2 nM) with acceptable BuChE inhibition activity (IC_50_ = 24 nM, more than 100 times more active than the standard drug), represented an additional advantage through reducing β-amyloid self and AChE-induced aggregation more active than the standard drug and also revealed higher neuroprotective activity against H_2_O_2_-induced cell death in PC12 cells and against amyloid toxic effects in SH-SY5Y cells than the reference drugs. All the results suggest that the new designed hybrids of coumarin and pyridinium parts could be considered as promising multifunctional agents for further developments in the field of anti-Alzheimer drugs.

## 4 Experimental

### 4.1 Chemistry

All commercially available chemicals were purchased from Merck, Sigma, and across without further purification. FT-IR spectra were run on a Bruker Equinox 55 spectrometer. ^1^H NMR and ^13^C NMR were recorded on Brucker 400 MHz instrument with frequencies of 400 and 100 MHz, respectively. DMSO was used as a solvent for NMR analyses and tetramethylsilane was employed as an internal standard. The chemical shifts (δ) and coupling constants (*J*) were expressed in parts per million and Hertz, respectively. Melting points were measured by the Buchi melting point B-540 B.V.CHI device. Analytical thin-layer chromatography (TLC) was performed on aluminum plates precoated with silica gel 60F-254 as the adsorbent. Mass spectra were obtained by HP Agilent Technologies 5973 at ionization potential of 70 eV. Elemental analyses were performed with CHNS-varioEL.

#### 4.1.1 General Procedure for the Synthesis of Compound **1**

The coumarin derivatives (**1a–f**) were synthesized via the Perkin–Oglialoro reaction according to the previously reported procedure (Abdshahzadeh et al., 2019). A solution containing anhydrous NaOAc (2 mmol), 4-Hydroxyphenylacetic acid (1 mmol), and salicylaldehyde (1 mmol) in Ac_2_O (1 ml) was refluxed for 2 h. After completion of the reaction, the mixture was cooled, neutralized with an aqueous NaHCO_3_ solution, filtered, and washed with distilled water. The compounds were used in the next step without further purification.

#### 4.1.2 General Procedure for the Synthesis of Intermediate **2**

Preparation of intermediate **2** was carried out according to the previously reported method (Hirbod et al., 2017). In brief, to a solution of 3-arylcoumarin (1 mmol) and anhydrous K_2_CO_3_ (2 mmol) in acetone (3 ml), corresponding dibromoalkane (10 mmol) was added and the solution was refluxed for 4 h until the starting material disappeared (monitored by TLC). The solvent was removed under vacuum, hexane was added to the residue, and the product was filtered off and used in the next step without further purification.

#### 4.1.3 General Procedure for the Synthesis of Compounds **3a–q**

To a solution containing compound **2** (0.25 mmol), the pyridine derivative (3.1 ml) was added. The mixture was stirred at 80°C for 24 h. The completion of the reaction was monitored by TLC. The mixture was cooled to room temperature, diethyl ether (10 ml) was added, and the mixture was cooled in the refrigerator for 2 h. The obtained solid was finally filtered and dried to obtain **3a–q**.

##### 4.1.3.1 1-(3-(4-(2-Oxo-2H-chromen-3-yl)phenoxy)propyl)pyridinium Bromide **3a**

Yield 91%; White solid; m.p. 280–282°C; FT-IR (ATR, cm^−1^) υ_max_: 3417, 2943, 1718, 1606, 1511, 1250, 1182, 1099, and 772; ^1^H NMR (400 MHz, DMSO-*d*_*6*_)/δ ppm: 9.18 (d, *J* = 4.0 Hz, 2H), 8.64 (t, *J* = 5.2 Hz, 1H), 8.18–8.19 (m, 3H), 7.77 (d, *J* = 5.6 Hz, 1H), 7.67 (d, *J* = 6.0 Hz, 2H), 7.59 (t, *J* = 5.6 Hz, 1H), 7.42 (d, *J* = 6.4 Hz, 1H), 7.38 (t, *J* = 5.2 Hz, 1H), 6.87 (d, *J* = 6.0 Hz, 2H), 4.84 (t, *J* = 4.0 Hz, 2H), 4.15 (t, *J* = 4.0 Hz, 2H), 2.35 (m, 2H); ^13^C NMR (100 MHz, DMSO-*d*_*6*_)/δ ppm: 159.8, 158.2, 152.7, 145.6, 145.1, 139.2, 131.4, 128.4, 127.9, 127.2, 126.3, 124.6, 119.6, 115.8, 114.0, 65.0, 58.9, 29.9; MS m/z: 438.3 (M^+^), 278.1 (C_18_H_15_O_3_^•^), 238.1 (C_15_H_9_O_3_^•^), 79.1 (C_5_H_5_N^•+^); Anal. Calcd. for C_23_H_20_BrNO_3_ (438.31): C, 63.02; H, 4.60; N, 3.20. Found: C, 62.96; H, 4.71; N, 3.00.

##### 4.1.3.2 1-(3-(4-(6-Methoxy-2-oxo-2H-chromen-3-yl)phenoxy)propyl)pyridinium Bromide **3b**

Yield 94%; White solid; m.p. 196–198°C; FT-IR (ATR cm^−1^) υ_max_: 3417, 3025, 1706, 1605, 1576, 1248, 1112, 1050, 771; ^1^H NMR (400 MHz, DMSO-*d*_*6*_)/δ ppm: 9.18 (d, *J* = 4.8 Hz, 2H), 8.64 (t, *J* = 5.2 Hz, 1H), 8.14–8.21 (m, 3H), 7.65 (d, *J* = 6.8 Hz, 2H), 7.36 (d, *J* = 7.2 Hz, 1H), 7.32 (s, 1H), 7.18 (d, *J* = 5.2 Hz, 1H), 6.87 (d, *J* = 6.8 Hz, 2H), 4.84 (t, *J* = 5.2 Hz, 2H), 4.15 (t, *J* = 4.0 Hz, 2H), 3.81 (s, OCH_3_, 3H), 2.20 (m, 2H); ^13^C NMR (100 MHz, DMSO-*d*_*6*_)/δ ppm: 159.9, 158.2, 155.6, 147.1, 145.1, 139.1, 129.8, 128.2, 127.9, 127.2, 126.5, 120.0, 118.8, 116.9, 114.2, 114.0, 110.6, 65.0, 58.9, 55.7, 29.9; MS m/z: 468.3 (M+), 388.1 (C_24_H_22_NO_4_^2•+^), 268.1 (C_16_H_11_O_4_^•^), 139.1 (C_8_H_11_NO^•+^), 79.1 (C_5_H_5_N^•+^); Anal. Calcd. for C_24_H_22_BrNO_4_ (468.34): C, 61.55; H, 4.73; N, 2.99. Found: C, 61.34; H, 4.81; N, 2.97.

##### 4.1.3.3 1-(3-(4-(7-Methoxy-2-oxo-2H-chromen-3-yl)phenoxy)propyl)pyridinium Bromide **3c**

Yield 88%; Cream solid; m.p. 256–258°C; FT-IR (ATR cm^−1^) υ_max_: 3466, 3017, 1714, 1604, 1510, 1250, 1118, 1049, 771; ^1^H NMR (400 MHz, DMSO-*d*_*6*_)/δ ppm: 9.17 (d, *J* = 4.4 Hz, 2H), 8.63 (t, *J* = 6.4 Hz, 1H), 8.13–8.17 (m, 3H), 7.64 (m, 3H), 6.97–7.02 (m, 2H), 6.84 (m, 2H), 4.84 (t, *J* = 4.0 Hz, 2H), 4.14 (t, *J* = 4.0 Hz, 2H), 3.86 (s, OCH_3_, 3H), 2.34 (m, 2H); ^13^C NMR (100 MHz, DMSO-*d*_*6*_)/δ ppm: 162.4, 160.1, 157.9, 154.5, 145.6, 145.3, 144.9, 129.7, 129.4, 128.0, 127.9, 127.4, 113.9, 113.1, 100.1, 95.1, 65.3, 58.9, 56.4, 26.7.

##### 4.1.3.4 1-(3-(4-(8-Methoxy-2-oxo-2H-chromen-3-yl)phenoxy)propyl)pyridinium Bromide **3d**

Yield 89%; Cream solid; m.p. 209–211°C; FT-IR (ATR cm^−1^) υ_max_: 3419, 3013, 1719, 1606, 1510, 1249, 1177, 1095, 772; ^1^H NMR (400 MHz, DMSO-*d*_*6*_)/δ ppm: 9.18 (d, *J* = 4.8 Hz, 2H), 8.63 (t, *J* = 4.0 Hz, 1H), 8.17–8.21 (m, 3H), 7.67 (d, *J* = 6.8 Hz, 2H), 7.28–7.31 (m, 3H), 6.86 (d, *J* = 6.8 Hz, 2H), 4.46 (t, *J* = 4.0 Hz, 2H), 4.10 (t, *J* = 4.0 Hz, 2H), 3.92 (s, OCH_3_, 3H), 2.25 (m, 2H); ^13^C NMR (100 MHz, DMSO-*d*_*6*_)/δ ppm: 159.5, 158.2, 146.2, 145.1, 145.0, 142.0, 139.4, 129.8, 127.9, 127.1, 126.3, 124.5, 120.1, 119.7, 114.0, 113.6, 65.0, 58.9, 56.1, 29.9.

##### 4.1.3.5 1-(3-(4-(6-Bromo-2-oxo-2H-chromen-3-yl)phenoxy)propyl)pyridinium Bromide **3e**

Yield 94%; White solid; m.p. 232–234°C; FT-IR (ATR cm^−1^) υ_max_: 3445, 3018, 1719, 1606, 1511, 1251, 1098, 770; ^1^H NMR (400 MHz, DMSO-*d*_*6*_)/δ ppm: 9.20 (d, *J* = 5.6 Hz, 2H), 8.66 (t, *J* = 7.6 Hz, 1H), 8.17–8.22 (m, 3H), 8.03 (s, 1H), 7.77 (d, *J* = 7.6 Hz, 1H), 7.68 (d, *J* = 8.4 Hz, 2H), 7.43 (d, *J* = 8.8 Hz, 1H), 6.91 (d, *J* = 8.4 Hz, 2H), 4.86 (t, *J* = 6.4 Hz, 2H), 4.18 (t, *J* = 6.4 Hz, 2H), 2.52 (m, 2H); ^13^C NMR (100 MHz, DMSO-*d*_*6*_)/δ ppm: 159.8, 158.9, 152.2, 146.1, 145.5, 138.3, 134.1, 130.8, 130.3, 128.4, 127.8, 127.3, 122.0, 118.6, 116.5, 114.6, 65.5, 59.4, 30.3.

##### 4.1.3.6 1-(3-(4-(6-Nitro-2-oxo-2H-chromen-3-yl)phenoxy)propyl)pyridinium Bromide **3f**

Yield 93%; Cream solid; m.p. 240–243°C; FT-IR (ATR cm^−1^) υ_max_: 3444, 3011, 1742, 1607, 1510, 1231, 769; ^1^H NMR (400 MHz, DMSO-*d*_*6*_)/δ ppm: 9.18 (d, *J* = 4.4 Hz, 2H), 8.74 (s, 1H), 8.63 (t, *J* = 6.0 Hz, 1H), 8.36–8.41 (m, 2H), 8.18 (t, *J* = 5.2 Hz, 2H), 7.64–7.68 (m, 3H), 6.89 (d, *J* = 6.8 Hz, 2H), 4.83 (t, *J* = 5.2 Hz, 2H), 4.16 (t, *J* = 4.0 Hz, 2H), 2.22 (m, 2H); ^13^C NMR (100 MHz, DMSO-*d*_*6*_)/δ ppm: 159.5, 158.9, 158.6, 156.3, 145.6, 145.1, 143.6, 129.9, 128.0, 128.0, 126.4, 125.8, 120.0, 117.3, 114.1, 65.0, 58.9, 29.9; MS m/z: 483.3 (M^+^), 323.2 (C_18_H_14_NO_5_^•^), 282.1 (C_15_H_8_NO_5_^•^), 79.2 (C_5_H_5_N^•+^); Anal. Calcd. for C_23_H_19_BrN_2_O_5_ (483.31): C, 57.16; H, 3.96; N, 5.80. Found: C, 57.01; H, 3.98; N, 5.71.

##### 4.1.3.7 1-(4-(4-(2-Oxo-2H-chromen-3-yl)phenoxy)butyl)pyridinium Bromide **3g**

Yield 93%; Pale yellow solid; m.p. 180–185°C; FT-IR (ATR cm^−1^) υ_max_: 3413, 3013, 1708, 1607, 1510, 1250, 1176, 1098, 773; ^1^H NMR (400 MHz, DMSO-*d*_*6*_)/δ ppm: 9.17 (d, *J* = 4.4 Hz, 2H), 8.63 (t, *J* = 6.0 Hz, 1H), 8.18–8.20 (m, 3H), 7.77 (d, *J* = 6.0 Hz, 1H), 7.69 (d, *J* = 6.8 Hz, 2H), 7.59 (t, *J* = 6.0 Hz, 1H), 7.42 (d, *J* = 6.4 Hz, 1H), 7.37 (t, *J* = 5.6 Hz, 1H), 7.01 (d, *J* = 6.8 Hz, 2H), 4.72 (t, *J* = 6.0 Hz, 2H), 4.07 (t, *J* = 4.4 Hz, 2H), 2.09–2.12 (m, 2H), 1.75–1.77 (m, 2H). ^13^C NMR (100 MHz, DMSO-*d*_*6*_)/δ ppm: 159.9, 158.7, 152.7, 145.5, 144.8, 139.1, 131.3, 129.8, 128.4, 128.1, 126.9, 126.3, 124.6, 119.6, 115.8, 114.2, 66.8, 60.4, 27.7, 25.2; MS m/z: 452.3 (M^+^), 238.1 (C_15_H_9_O_3_^•^), 135.1 (C_9_H_13_N^•+^), 79.1 (C_5_H_5_N^•+^), 55.1 (C_4_H_8_^2•^); Anal. Calcd. for C_24_H_22_BrNO_3_ (452.34): C, 63.73; H, 4.90; N, 3.10. Found: C, 63.59; H, 4.98; N, 2.95.

##### 4.1.3.8 1-(4-(4-(6-Methoxy-2-oxo-2H-chromen-3-yl)phenoxy)butyl)pyridinium Bromide **3h**

Yield 92%; Cream solid; m.p. 189–192°C; FT-IR (ATR cm^−1^) υ_max_: 3444, 2946, 1710, 1606, 1578, 1254, 1104, 1023, 770; ^1^H NMR (400 MHz, DMSO-d_6_)/δ ppm: 9.14 (d, *J* = 4.0 Hz, 2H), 8.61 (t, *J* = 5.6 Hz, 1H), 8.14–8.18 (m, 3H), 7.67 (d, *J* = 6.4 Hz, 2H), 7.37 (d, *J* = 6.8 Hz, 1H), 7.31 (s, 1H), 7.19 (d, *J* = 6.4 Hz, 1H), 7.01 (d, *J* = 6.4 Hz, 2H), 4.70 (t, *J* = 5.6 Hz, 2H), 4.07 (t, *J* = 4.0 Hz, 2H), 3.81 (s, OCH_3_, 3H), 2.1 (m, 2H), 1.77 (m, 2H); ^13^C NMR (100 MHz, DMSO-*d*_*6*_)/δ ppm: 159.8, 158.6, 155.6, 148.1, 144.9, 144.4, 142.1, 139.2, 129.8, 128.1, 126.5, 119.9, 118.2, 116.9, 114.2, 110.4, 66.8, 60.7, 55.6, 27.5, 25.0.

##### 4.1.3.9 1-(4-(4-(7-Methoxy-2-oxo-2H-chromen-3-yl)phenoxy)butyl)pyridinium Bromide **3i**

Yield 90%; Brown solid; m.p. 186–188°C; FT-IR (ATR cm^−1^) υ_max_: 3479, 3013, 1719, 1606, 1510, 1250, 1117, 1096, and 773; ^1^H NMR (400 MHz, DMSO-*d*_*6*_)/δ ppm: 9.16 (d, *J* = 4.0 Hz, 2H), 8.62 (t, *J* = 8.0 Hz, 1H), 8.14–8.18 (m, 3H), 7.67 (m, 3H), 6.98–7.01 (m, 4H), 4.71 (t, *J* = 4.0 Hz, 2H), 4.06 (t, *J* = 4.0 Hz, 2H), 3.86 (s, OCH_3_, 3H), 2.11 (m, 2H), and 1.76 (m, 2H); ^13^C NMR (100 MHz, DMSO-*d*_*6*_)/δ ppm: 166.05, 162.61, 152.72, 145.59, 144.73, 139.92, 131.39, 129.62, 128.38, 128.08, 126.59, 126.35, 123.07, 119.97, 114.22, 112.41, 66.84, 60.81, 55.67, 55.35, 27.48, 25.44.

##### 4.1.3.10 1-(4-(4-(8-Methoxy-2-oxo-2H-chromen-3-yl)phenoxy)butyl)pyridinium Bromide **3j**

Yield 90%; Pale yellow solid; m.p. 105–107°C; FT-IR (ATR cm^−1^) υ_max_: 3416, 3012, 2940, 1719, 1606, 1574, 1276, 1177, 1095, and 773; ^1^H NMR (400 MHz, DMSO-*d*_*6*_)/δ ppm: 9.17 (d, *J* = 4.0 Hz, 2H), 8.62 (t, *J* = 6.0 Hz, 1H), 8.17 (m, 3H), 7.68–7.70 (m, 2H), 7.28–7.30 (m, 3H), 7.00 (d, *J* = 6.4 Hz, 2H), 4.70 (t, *J* = 5.2 Hz, 2H), 4.06 (t, *J* = 4.0 Hz, 2H), 3.91 (s, OCH_3_, 3H), 2.11 (m, 2H), and 1.76 (m, 2H); ^13^C NMR (100 MHz, DMSO-d_6_)/δ ppm: 159.6, 158.7, 146.2, 146.2, 145.5, 144.6, 141.9, 139.2, 129.9, 129.6, 128.2, 126.8, 120.4, 124.5, 120.1, 114.2, 66.8, 60.4, 55.9, 27.7, 25.2.

##### 4.1.3.11 1-(4-(4-(6-Bromo-2-oxo-2H-chromen-3-yl)phenoxy)butyl)pyridinium Bromide **3k**

Yield 94%; Cream solid; m.p. 256–258°C; FT-IR (ATR cm^−1^) υ_max_: 3412, 2943, 1727, 1606, 1511, 1251, 1182, 1098, and 774; ^1^H NMR (400 MHz, DMSO-d_6_)/δ ppm: 9.21 (d, *J* = 5.2 Hz, 2H), 8.65 (t, *J* = 7.6 Hz, 1H), 8.18–8.23 (m, 3H), 8.03 (s, 1H), 7.75 (d, *J* = 8.8 Hz, 1H), 7.69 (d, *J* = 8.4 Hz, 2H), 7.41 (d, *J* = 8.8 Hz, 1H), 7.04 (d, *J* = 8.4 Hz, 2H), 4.75 (t, *J* = 7.2 Hz, 2H), 4.10 (t, *J* = 6.0 Hz, 2H), 2.10–2.15 (m, 2H), and 1.77–1.81 (m, 2H); ^13^C NMR (100 MHz, DMSO-*d*_*6*_)/δ ppm: 159.8, 158.4, 152.2, 146.0, 145.3, 138.1, 134.0, 130.8, 130.3, 128.6, 127.8, 127.0, 122.0, 118.5, 116.5, 114.7, 67.4, 60.9, 28.2, 25.6.

##### 4.1.3.12 1-(4-(4-(6-Nitro-2-oxo-2H-chromen-3-yl)phenoxy)butyl)pyridinium Bromide **3l**

Yield 93%; Yellow solid; m.p. 223–226°C; FT-IR (ATR cm^−1^) υ_max_: 3407, 2943, 1724, 1607, 1513, 1252, 1182, 1095, and 773; ^1^H NMR (400 MHz, DMSO-*d*_*6*_)/δ ppm: 9.17 (d, *J* = 4.4 Hz, 2H), 8.74 (s, 1H), 8.62 (t, *J* = 6.0 Hz, 1H), 8.37–8.39 (m, 2H), 8.19 (t, *J* = 5.6 Hz, 2H), 7.69 (d, *J* = 6.8 Hz, 2H), 7.64 (d, *J* = 7.2 Hz, 1H), 7.04 (d, *J* = 6.8 Hz, 2H), 4.72 (t, *J* = 5.6 Hz, 2H), 4.08 (t, *J* = 4.8 Hz, 2H), 2.10–2.13 (m, 2H), and 1.75–1.78 (m, 2H); ^13^C NMR (100 MHz, DMSO-*d*_*6*_)/δ ppm: 159.1, 158.9, 156.3, 145.5, 144.8, 143.5, 137.8, 129.9, 128.1, 128.0, 126.4, 126.1, 125.8, 124.1, 120.0, 117.3, 114.3, 66.9, 60.4, and 27.7, 25.2; MS m/z: 497.3 (M^+^), 283.1 (C_15_H_8_NO_5_^•^), 135.1 (C_9_H_13_N^•+^), 55.2 (C_4_H_8_^2•^); Anal. Calcd. for C_24_H_21_BrN_2_O_5_ (497.34): C, 57.96; H, 4.26; N, 5.63. Found: C, 57.73; H, 4.35; N, 5.58.

##### 4.1.3.13 1-(5-(4-(2-Oxo-2H-chromen-3-yl)phenoxy)pentyl)pyridinium Bromide **3m**

Yield 94%; White solid; m.p. 100–103°C; FT-IR (ATR cm^−1^) υ_max_: 3443, 2943, 1695, 1606, 1512, 1280, 1124, 1050, and 766; ^1^H NMR (400 MHz, DMSO-*d*_*6*_)/δ ppm: 9.12 (d, *J* = 4.4 Hz, 2H), 8.62 (t, *J* = 6.0 Hz, 1H), 8.16–8.19 (m, 3H), 7.77 (d, *J* = 6.0 Hz, 1H), 7.69 (d, *J* = 6.8 Hz, 2H), 7.60 (t, *J* = 6.0 Hz, 1H), 7.42 (d, *J* = 6.4 Hz, 1H), 7.37 (t, *J* = 6.0 Hz, 1H), 7.00 (d, *J* = 6.8 Hz, 2H), 4.64 (t, *J* = 6.0 Hz, 2H), 4.03 (t, *J* = 4.8 Hz, 2H), 2.00–2.02 (m, 2H), 1.77–1.80 (m, 2H), 1.43–1.46 (m, 2H); ^13^C NMR (100 MHz, DMSO-*d*_*6*_)/δ ppm: 158.9, 158.7, 152.7, 145.5, 144.8, 138.8, 131.3, 129.8, 128.4, 128.1, 126.9, 126.3, 124.6, 119.6, 115.8, 114.2, 66.8, 60.6, 30.4, 28.6, 22.0.

##### 4.1.3.14 1-(5-(4-(8-Methoxy-2-oxo-2H-chromen-3-yl)phenoxy)pentyl)pyridinium Bromide **3n**

Yield 92%; Cream solid; m.p. 234–235°C; FT-IR (ATR cm^−1^) υ_max_: 3357, 2941, 1697, 1606, 1278, 1098, 1050, 775; ^1^H NMR (400 MHz, DMSO-*d*_*6*_)/δ ppm: 9.14 (d, *J* = 4.4 Hz, 2H), 8.62 (t, *J* = 6.0 Hz, 1H), 8.16–8.19 (m, 3H), 7.70 (d, *J* = 6.8 Hz, 2H), 7.28–7.31 (m, 3H), 6.98 (d, *J* = 6.8 Hz, 2H), 4.66 (t, *J* = 5.6 Hz, 2H), 4.03 (t, *J* = 4.8 Hz, 2H), 3.92 (s, OCH_3_, 3H), 1.99–2.02 (m, 2H), 1.77–1.80 (m, 2H), and 1.45–1.46 (m, 2H); ^13^C NMR (100 MHz, DMSO-*d*_*6*_)/δ ppm: 159.6, 158.9, 146.2, 145.5, 144.8, 141.9, 139.2, 129.8, 128.1, 126.7, 126.4, 124.5, 120.1, 119.6, 114.1, 113.5, 67.1, 60.6, 56.0, 30.4, 27.9, 22.0.

##### 4.1.3.15 1-(5-(4-(6-Nitro-2-oxo-2H-chromen-3-yl)phenoxy)pentyl)pyridinium Bromide **3o**

Yield 91%; Pale yellow solid; m.p. 286–288°C; FT-IR (ATR cm^−1^) υ_max_: 3419, 2944, 1730, 1607, 1512, 1250, 1096, 769; ^1^H NMR (400 MHz, DMSO-*d*_*6*_)/δ ppm: 9.13 (d, *J* = 4.4 Hz, 2H), 8.74 (s, 1H), 8.62 (t, *J* = 6.0 Hz, 1H), 8.38–8.40 (m, 2H), 8.18 (t, *J* = 5.6 Hz, 2H), 7.69 (d, *J* = 6.8 Hz, 2H), 7.64 (d, *J* = 7.2 Hz, 1H), 7.03 (d, *J* = 6.8 Hz, 2H), 4.66 (t, *J* = 6.0 Hz, 2H), 4.04 (t, *J* = 5.2 Hz, 2H), 2.00–2.04 (m, 2H), 1.76–1.81 (m, 2H), 1.42–1.48 (m, 2H); ^13^C NMR (100 MHz, DMSO-*d*_*6*_)/δ ppm: 159.3, 158.9, 156.13, 145.5, 144.8, 143.5, 137.7, 129.9, 128.1, 126.0, 125.7, 124.1, 120.0, 117.3, 114.3, 67.2, 60.5, 30.4, 27.9, 22.0.

##### 4.1.3.16 1-(5-(4-(6-Bromo-2-oxo-2H-chromen-3-yl)phenoxy)pentyl)pyridinium Bromide **3p**

Yield 95%; Cream solid; m.p. 240–242°C; FT-IR (ATR cm^−1^) υ_max_: 3449, 2944, 1711, 1605, 1508, 1283, 1113, and 771; ^1^H NMR (400 MHz, DMSO-*d*_*6*_)/δ ppm: 9.21 (d, *J* = 5.6 Hz, 2H), 8.65 (t, *J* = 7.6 Hz, 1H), 8.19–8.22 (m, 3H), 8.02 (s, 1H), 7.75 (d, *J* = 8.4 Hz, 1H), 7.69 (d, *J* = 8.0 Hz, 2H), 7.41 (d, *J* = 8.8 Hz, 1H), 7.04 (d, *J* = 8.0 Hz, 2H), 4.71 (t, *J* = 6.8 Hz, 2H), 4.03 (t, *J* = 6.0 Hz, 2H), 2.01–2.09 (m, 2H), 1.78–1.81 (m, 2H), 1.45–1.48 (m, 2H); ^13^C NMR (100 MHz, DMSO-*d*_*6*_)/δ ppm: 159.8, 158.4, 152.2, 146.0, 145.3, 138.1, 134.0, 130.8, 130.3, 128.6, 127.8, 127.0, 122.0, 118.5, 116.5, 114.7, 67.4, 60.9, 30.9, 28.2, 25.6.

##### 4.1.3.17 1-(5-(4-(6-Bromo-2-oxo-2H-chromen-3-yl)phenoxy)pentyl)-5-ethyl-2-methylpyridin-1-ium Bromide **3q**

Yield 54%; Yellow oil; FT-IR (KBr cm^−1^) υ_max_: 3394, 2921, 1720, 1613, 1530, 1467, 1252, 1112, 828; ^1^H NMR (400 MHz, DMSO-*d*_*6*_)/δ ppm: 8.98 (s, 1H), 8.40 (d, *J* = 8.8 Hz, 1H), 8.18 (s, 1H), 8.04 (d, *J* = 2.4 Hz, 1H), 7.99 (d, *J* = 8.4 Hz, 1H), 7.76 (d, *J* = 6.8 Hz,1H), 7.70 (d, *J* = 8.8 Hz, 2H), 7.42 (d, *J* = 8.8 Hz, 1H), 7.04 (d, *J* = 8.8 Hz, 2H), 4.57 (t, *J* = 7.6 Hz, 2H), 4.08 (t, *J* = 6.4 Hz, 2H), 2.82 (s, 3H), 2.78–2.80 (m, 2H), 1.85–1.96 (m, 2H), and 1.25–1.28 (m, 4H); 1.04–1.06 (m, 3H); ^13^C NMR (100 MHz, DMSO-*d*_*6*_)/δ ppm: 159.8, 158.4, 152.2, 146.0, 145.3, 138.1, 134.0, 130.8, 130.3, 128.6, 127.8, 127.0, 122.0, 118.5, 116.5, 114.7, 67.4, 60.9, 30.9, 28.2, and 25.6; ^13^C NMR (100 MHz, DMSO-*d*_*6*_)/δ ppm: 159.9, 159.6, 152.8, 152.2, 145.1, 144.6, 142.0, 138.1, 134.0, 130.8, 130.3, 129.9, 127.9, 126.9, 122.0, 118.6, 116.5, 114.7, 67.7, 57.5, 29.6, 28.5, 25.1, 22.9, 19.5, 14.8.

#### 4.1.4 General Procedure for the Synthesis of Compound **3r–t**

A mixture of compound **2** (1 mmol) and 4-dimethylaminopyridine (1.5 mmol) in dry acetonitrile (5 ml) was stirred at 70°C for 24 h. After completion of the reaction (monitored by TLC), the mixture was cooled to room temperature and acetone (20 ml) was then added. The mixture was cooled in the refrigerator (5°C) overnight. The solid was filtered and crystalized using acetone.

##### 4.1.4.1 4-(Dimethylamino)-1-(3-(4-(6-bromo-2-oxo-2H-chromen-3-yl)phenoxy)pentyl)pyridin-1-ium Bromide **3r**

Yield 65%; Yellow solid; m.p. 146–148°C; FT-IR (ATR, cm^−1^) υ_max_: 3375, 3067, 2942, 1716, 1649, 1606, 1565, 1472, 1255, 1179, 1066, and 825; ^1^H NMR (400 MHz, DMSO-*d*_*6*_)/δ ppm: 8.41 (d, *J* = 7.2 Hz, 2H), 8.19 (s, 1H), 8.02 (s, 1H), 7.73 (d, *J* = 1.6 Hz, 1H), 7.69 (d, *J* = 8.4 Hz, 2H), 7.38 (d, *J* = 8.8 Hz,1H), 7.06 (d, *J* = 7.2 Hz, 2H), 7.00 (d, *J* = 8.4 Hz, 2H), 4.26 (t, *J* = 6.8 Hz, 2H), 4.02 (t, *J* = 6.0 Hz, 2H), 3.83 (s, 6H), 1.84–1.87 (m, 2H), 1.76–1.81 (m, 2H), and 1.41–1.42 (m, 2H); ^13^C NMR (100 MHz, DMSO-*d*_*6*_)/δ ppm: 159.8, 159.5, 158.4, 156.2, 152.1, 142.5, 138.1, 134.0, 130.8, 130.3, 127.7, 126.8, 122.0, 118.5, 116.5, 114.6, 108.1, 67.7, 56.9, 30.5, 28.4, 22.5.

##### 4.1.4.2 4-(Dimethylamino)-1-(3-(4-(2-oxo-2H-chromen-3-yl)phenoxy)propyl)pyridin-1-ium Bromide **3s**

Yield 60%; White solid; m.p. 233–235°C; FT-IR (ATR cm^−1^) υ_max_: 3370, 3057, 2883, 1715, 1645, 1607, 1568, 1509, 1255, 1177, 1115, 768; ^1^H NMR (400 MHz, DMSO-*d*_*6*_)/δ ppm: 8.43 (d, *J* = 7.6 Hz, 2H), 8.23 (s, 1H), 7.80 (d, *J* = 7.6 Hz, 1H), 7.70 (d, *J* = 8.8 Hz, 2H), 7.61 (t, *J* = 7.6 Hz,1H), 7.42 (d, *J* = 8.4 Hz, 1H), 7.37 (d, *J* = 7.6 Hz, 1H), 7.06 (d, *J* = 7.2 Hz, 2H), 6.98 (d, *J* = 8.8 Hz, 2H), 4.43 (t, *J* = 6.4 Hz, 2H), 4.08 (t, *J* = 5.6 Hz, 2H), 3.20 (s, 6H), 2.29–2.32 (m, 2H); ^13^C NMR (100 MHz, DMSO-*d*_*6*_)/δ ppm: 160.3, 158.9, 156.3, 153.1, 142.7, 139.7, 131.8, 130.3, 128.9, 127.5, 126.7, 125.0, 120.0, 116.2, 114.6, 108.1, 65.1, 54.7, 40.2, 30.1. MS m/z: 481.3 (M^+^), 388.1 (C_24_H_22_N_2_O_3_^•+^), 238.1 (C_15_H_9_O_3_^•^), 121.2 (C_7_H_10_N_2_^•+^); Anal. Calcd. for C_25_H_25_BrN_2_O_3_ (481.38): C, 62.38; H, 5.23; N, 5.82; O, 9.97. Found: C, 62.25; H, 5.42; N, 5.77.

##### 4.1.4.3 4-(Dimethylamino)-1-(3-(4-(6-nitro-2-oxo-2H-chromen-3-yl)phenoxy)propyl)pyridin-1-ium Bromide **3t**

Yield 72%; Yellow solid; m.p. 173–174°C; FT-IR (ATR cm^−1^) υ_max_: 3365, 1722, 1649, 1611, 1564, 1517, 1343, 1255, 1176, 1095, 942, 825; ^1^H NMR (400 MHz, DMSO-*d*_*6*_)/δ ppm: 8.76 (d, *J* = 2.8 Hz, 1H), 8.43 (d, *J* = 2.4 Hz, 1H), 8.40 (s, 1H), 8.37 (d, *J* = 7.2 Hz, 2H), 7.72 (d, *J* = 8.8 Hz,2H), 7.68 (d, *J* = 8.8 Hz, 1H), 7.11 (d, *J* = 7.6 Hz, 2H), 7.02 (d, *J* = 8.8 Hz, 2H), 4.41 (t, *J* = 6.8 Hz, 2H), 4.10 (t, *J* = 5.6 Hz, 2H), 3.21 (s, 6H), 2.30–2.33 (m, 2H); ^13^C NMR (100 MHz, DMSO-*d*_*6*_)/δ ppm: 159.4, 159.3, 156.8, 156.3, 144.1, 142.7, 138.3, 130.4, 128.5, 126.9, 126.3, 124.6, 120.5, 117.8, 114.7, 108.1, 65.1, 54.8, 40.2, 30.0.

### 4.2 Biological Assays

#### 4.2.1 Inhibitory Activity of the Target Compounds Against AChE and BChE

To evaluate the AChE and BuChE inhibitory activities, the Ellman’s method was employed (Ellman et al., 1961). AChE, BuChE, 5,5'-dithiobis (2-nitrobenzoic acid) (DTNB), acetyl- and butyrylthiocholine iodides were purchased from Sigma-Aldrich. Five different concentrations of the corresponding compounds in ethanol-DMSO (9:1) were prepared. For assay, the corresponding enzyme (5 IU/ml, in phosphate buffer, pH 8.0 containing 25% v/v of glycerol) was added to a 24-well plate containing PBS, the tested compound in different concentrations, and DTNB (0.01 M). After 3 min of incubation, the substrate solution (acetylthiocholine iodide or butyrylthiocholine iodide, 0.05 M) was added and then incubated for at least 1 min at 25°C. The absorbance was measured at 412 μM using a microplate reader (BioTek Synergy HT). The inhibition curve was obtained by plotting the percentage of enzyme activity (100% for the reference) versus the logarithm of the tested compound concentration. Results are reported as the mean ± SD for at least three different experiments.

#### 4.2.2 Neuroprotection Assay Against H_2_O_2_-Induced Cell Death in PC12 Cells

The cell viability was measured using MTT [3-(4,5-dimethylthiazol-2-yl)-2,5-diphenyl tetrazolium bromide] assay (Datki et al., 2003). PC12 cells (from the Iranian Biological Resource Center, IBRC) received different treatments, including no treatment (control), 150 μM H_2_O_2_ alone, or 150 μM H_2_O_2_ in combination with 0.1–50 μM of the tested compound. The cells were seeded at 1 × 104 cells/well and incubated at 37ºC under a 5% CO_2_ atmosphere for 24 h, then treated with the tested compound and incubated for 3 h. Next, the cells were exposed to H_2_O_2_ for 2 h again. The MTT solution (20 μl, 5 mg/ml) was next replaced with the medium and incubated for 4 h. 100 μl of DMSO was added to dissolve the formazan precipitate. Absorbance was then measured at 570 nm using a multi-mode plate reader (Biotek, Winooski, VT). Cell viability is expressed as a percentage relative to the untreated control.

#### 4.2.3 Inhibition of Self- and AChE-Induced Aβ_1-42_ Aggregation

Inhibitory properties of the compounds on self-induced and AChE-induced aggregation of amyloid-β protein 1–42 was determined using a thioflavin T (ThT)-based fluorescence assay with slight modifications (Levine, 1993). The ThT excitation/emission was measured at 448 nm/490 nm at 48 h using a SpectraMax^®^ Microplate Reader. Amyloid-β protein 1–42 (Sigma A9810) was dissolved in Phosphate Buffer Saline pH 7.4 (PBS, HyClone Thermo Scientific) containing ammonium hydroxide (1%). Aβ_1-42_ (50 μM) was incubated for 24 h at 37°C for prefibrillation.

To determine AChE-induced Aβ_1-42_ aggregation, Aβ_1-42_ (20 μl) ± human recombinant AChE (0.01 u/ml, Sigma C1682) were added to 450 ml of PBS buffer pH 7.4 including 0.15 M NaCl and 20 μM thioflavin T (ThT). The mixture was incubated at 37°C in the absence and presence of the compounds (100 μM) and the fluorescence intensities were determined. Due to the presence of the tested compounds, inhibition of self- or AChE-induced aggregation percent was determined by the following calculation: [100-((IFi/IFo) × 100)] where IFi and IFo are the fluorescence intensities obtained for Aβ ± AChE in the presence and in the absence of inhibitors.

#### 4.2.4 Cytotoxicity and Neuroprotection Assay Against Aβ_1-42_-Induced Cytotoxicity in SH-SY5Y Cells

Cell culture chemicals were purchased from Lonza, HyClone, or Thermo Scientific. Human neuroblastoma SH-SY5Y cells were maintained in Dulbecco’s modified Eagle’s medium (DMEM) supplemented with 10% FBS, 1% L-glutamine, and 1% antibiotic mix at 37°C in a humidified atmosphere containing 5% CO_2_. Cell viability was determined using a thiazolyl blue tetrazolium bromide [3-(4,5-dimethyl-2-thiazolyl)-2,5-diphenyl-2*H*-tetrazolium bromide] (MTT) assay (Datki et al., 2003). SH-SY5Y cells were seeded into 384-well plates at 3,000 cells per well and treated with novel compounds at a concentration of 1 μM, 3 h prior to the addition of Aβ_1–42_ (5 μM). After 24 h of incubation, 5 μl of the MTT reagent (5 mg/ml) was added to each well. 40 μl of DMSO was used to dissolve formazan crystals. Absorbance values were measured at 690 and 570 nm using a BMG Omega Fluorostar microplate reader.

#### 4.2.5 Docking Simulations

Molecular docking simulation was conducted using AutoDock 4.2 (Goodsell et al., 1996). The crystal structures of the studied targets, PDBID: 1EVE (Kryger et al., 1999) and 4BDS (Nachon et al., 2013) for AChE and BuChE, were retrieved from the RCSB protein data bank website. The reason for the selection of these codes was their high resolution, 2.50 and 2.10 Å for 1EVE and 4BDS, respectively. The 3D structures of the potent compounds (**3f** and **3t**) were prepared by Chem3D software. The analysis of docking results was performed by the Discovery Studio software. To validate our docking protocol, re-dock simulations were performed. The results showed that the RMSD values were 0.96 and 0.66 for AChE and BuChE targets, respectively, which confirmed the accuracy of the docking protocol.

## Data Availability

The original contributions presented in the study are included in the article/Supplementary Material; further inquiries can be directed to the corresponding authors.

## References

[B1] Abdpour S. Jalili-Baleh L. Nadri H. Forootanfar H. Bukhari S. N. A. Ramazani A. (2021). Chromone Derivatives Bearing Pyridinium Moiety as Multi-Target-Directed Ligands against Alzheimer’s Disease. Bioorg. Chem. 110, 104750. 10.1016/j.bioorg.2021.104750 33691251

[B2] Abdshahzadeh H. Golshani M. Nadri H. Saberi Kia I. Abdolahi Z. Forootanfar H. (2019). 3‐Aryl Coumarin Derivatives Bearing Aminoalkoxy Moiety as Multi‐Target‐Directed Ligands against Alzheimer's Disease. Chem. Biodivers 16, e1800436. 10.1002/cbdv.201800436 30957958

[B3] Abu-Aisheh M. N. Al-Aboudi A. Mustafa M. S. El-Abadelah M. M. Ali S. Y. Ul-Haq Z. (2019). Coumarin Derivatives as Acetyl-And Butyrylcholinestrase Inhibitors: An *In Vitro*, Molecular Docking, and Molecular Dynamics Simulations Study. Heliyon 5, e01552. 10.1016/j.heliyon.2019.e01552 31183424 PMC6488543

[B4] Alcaro S. Bolognesi M. L. García-Sosa A. T. Rapposelli S. (2019). Multi-target-directed Ligands (MTDL) as Challenging Research Tools in Drug Discovery: From Design to Pharmacological Evaluation. Front. Chem. 7, 71. 10.3389/fchem.2019.00071 30834243 PMC6387964

[B5] Altaf A. A. Shahzad A. Gul Z. Khan S. A. Badshah A. Tahir M. N. (2015). A Review on the Medicinal Importance of Pyridine Derivatives. J. Chem. 2015, 1–5. 10.1155/2015/913435

[B6] Anand P. Singh B. Singh N. (2012). A Review on Coumarins as Acetylcholinesterase Inhibitors for Alzheimer's Disease. Bioorg. Med. Chem. 20, 1175–1180. 10.1016/j.bmc.2011.12.042 22257528

[B7] Bagheri S. M. Khoobi M. Nadri H. Moradi A. Emami S. Jalili-Baleh L. (2015). Synthesis and Anticholinergic Activity of 4‐hydroxycoumarin Derivatives Containing Substituted Benzyl‐1, 2, 3‐triazole Moiety. Chem. Biol. Drug Des. 86, 1215–1220. 10.1111/cbdd.12588 26010139

[B8] Bilgin H. M. Atmaca M. Deniz Obay B. Özekinci S. Taşdemir E. Ketani A. (2011). Protective Effects of Coumarin and Coumarin Derivatives against Carbon Tetrachloride-Induced Acute Hepatotoxicity in Rats. Exp. Toxicol. Pathol. 63, 325–330. 10.1016/j.etp.2010.02.006 20207117

[B9] Bolognesi M. L. Bartolini M. Tarozzi A. Morroni F. Lizzi F. Milelli A. (2011). Multitargeted Drugs Discovery: Balancing Anti-amyloid and Anticholinesterase Capacity in a Single Chemical Entity. Bioorg. Med. Chem. Lett. 21, 2655–2658. 10.1016/j.bmcl.2010.12.093 21236667

[B10] Canning C. Sun S. Ji X. Gupta S. Zhou K. (2013). Antibacterial and Cytotoxic Activity of Isoprenylated Coumarin Mammea A/AA Isolated from Mammea Africana. J. Ethnopharmacol. 147, 259–262. 10.1016/j.jep.2013.02.026 23466248

[B11] Cavalli A. Bolognesi M. L. Minarini A. Rosini M. Tumiatti V. Recanatini M. (2008). Multi-target-directed Ligands to Combat Neurodegenerative Diseases. J. Med. Chem. 51, 347–372. 10.1021/jm7009364 18181565

[B12] Chen W.-W. Zhang X. Huang W.-J. (2016). Role of Physical Exercise in Alzheimer's Disease. Biomed. Rep. 4, 403–407. 10.3892/br.2016.607 27073621 PMC4812200

[B13] Chen R. Chan P.-T. Chu H. Lin Y.-C. Chang P.-C. Chen C.-Y. (2017). Treatment Effects between Monotherapy of Donepezil versus Combination with Memantine for Alzheimer Disease: a Meta-Analysis. PLoS One 12, e0183586. 10.1371/journal.pone.0183586 28827830 PMC5565113

[B14] Choubdar N. Golshani M. Jalili-Baleh L. Nadri H. Küçükkilinç T. T. Ayazgök B. (2019). New Classes of Carbazoles as Potential Multi-Functional Anti-alzheimer's Agents. Bioorg. Chem. 91, 103164. 10.1016/j.bioorg.2019.103164 31398601

[B15] Datki Z. Juhász A. Gálfi M. Soós K. Papp R. Zádori D. (2003). Method for Measuring Neurotoxicity of Aggregating Polypeptides with the MTT Assay on Differentiated Neuroblastoma Cells. Brain Res. Bull. 62, 223–229. 10.1016/j.brainresbull.2003.09.011 14698355

[B16] Davidsson P. Blennow K. Andreasen N. Eriksson B. Minthon L. Hesse C. (2001). Differential Increase in Cerebrospinal Fluid-Acetylcholinesterase after Treatment with Acetylcholinesterase Inhibitors in Patients with Alzheimer's Disease. Neurosci. Lett. 300, 157–160. 10.1016/S0304-3940(01)01586-5 11226635

[B17] Du H. Liu X. Xie J. Ma F. (2019). Novel Deoxyvasicinone–Donepezil Hybrids as Potential Multitarget Drug Candidates for Alzheimer’s Disease. ACS Chem. Neurosci. 10, 2397–2407. 10.1021/acschemneuro.8b00699 30720268

[B18] Ellman G. L. Courtney K. D. Andres V. Jr Featherstone R. M. (1961). A New and Rapid Colorimetric Determination of Acetylcholinesterase Activity. Biochem. Pharmacol. 7, 88–95. 10.1016/0006-2952(61)90145-9 13726518

[B19] González J. F. Alcántara A. R. Doadrio A. L. Sánchez-Montero J. M. (2019). Developments with Multi-Target Drugs for Alzheimer’s Disease: an Overview of the Current Discovery Approaches. Expert Opin. drug Discov. 14, 879–891. 10.1080/17460441.2019.1623201 31165654

[B20] Goodsell D. S. Morris G. M. Olson A. J. (1996). Automated Docking of Flexible Ligands: Applications of AutoDock. J. Mol. Recognit. 9, 1–5. 10.1002/(sici)1099-1352(199601)9:1<1::aid-jmr241>3.0.co;2-6 8723313

[B21] Hirbod K. Jalili-Baleh L. Nadri H. Ebrahimi S. E. S. Moradi A. Pakseresht B. (2017). Coumarin Derivatives Bearing Benzoheterocycle Moiety: Synthesis, Cholinesterase Inhibitory, and Docking Simulation Study. Iran. J. Basic Med. Sci. 20, 631–638. 10.22038/IJBMS.2017.8830 28868119 PMC5569448

[B22] Hopkins A. L. (2008). Network Pharmacology: the Next Paradigm in Drug Discovery. Nat. Chem. Biol. 4, 682–690. 10.1038/nchembio.118 18936753

[B23] Inestrosa N. C. Sagal J. P. Colombres M. (2005). Acetylcholinesterase Interaction with Alzheimer Amyloid β. Alzheimer’s Dis. 38, 299–317. 10.1007/0-387-23226-5_15 15709485

[B24] Jeremic D. Jiménez-Díaz L. Navarro-López J. D. (2021). Past, Present and Future of Therapeutic Strategies against Amyloid-β Peptides in Alzheimer’s Disease: a Systematic Review. Ageing Res. Rev. 72, 101496. 10.1016/j.arr.2021.101496 34687956

[B25] Kapková P. Alptüzün V. Frey P. Erciyas E. Holzgrabe U. (2006). Search for Dual Function Inhibitors for Alzheimer’s Disease: Synthesis and Biological Activity of Acetylcholinesterase Inhibitors of Pyridinium-type and Their Aβ Fibril Formation Inhibition Capacity. Bioorg. Med. Chem. 14, 472–478. 10.1016/j.bmc.2005.08.034 16198581

[B26] Koyiparambath V. P. Prayaga Rajappan K. Rangarajan T. M. Al‐Sehemi A. G. Pannipara M. Bhaskar V. (2021). Deciphering the Detailed Structure–Activity Relationship of Coumarins as Monoamine Oxidase Enzyme Inhibitors—An Updated Review. Chem. Biol. Drug Des. 98, 655–673. 10.1111/cbdd.13919 34233082

[B27] Kryger G. Silman I. Sussman J. L. (1999). Structure of Acetylcholinesterase Complexed with E2020 (Aricept®): Implications for the Design of New Anti-alzheimer Drugs. Structure 7, 297–307. 10.1016/s0969-2126(99)80040-9 10368299

[B28] Levine H. iii (1993). Thioflavine T Interaction with Synthetic Alzheimer's Disease β‐amyloid Peptides: Detection of Amyloid Aggregation in Solution. Protein Sci. 2, 404–410. 10.1002/pro.5560020312 8453378 PMC2142377

[B29] Li G. Hong G. Li X. Zhang Y. Xu Z. Mao L. (2018). Synthesis and Activity towards Alzheimer's Disease *In Vitro*: Tacrine, Phenolic Acid and Ligustrazine Hybrids. Eur. J. Med. Chem. 148, 238–254. 10.1016/j.ejmech.2018.01.028 29466774

[B30] Li K. Jiang Y. Li G. Liu T. Yang Z. (2020). Novel Multitarget Directed Tacrine Hybrids as Anti-alzheimer’s Compounds Improved Synaptic Plasticity and Cognitive Impairment in APP/PS1 Transgenic Mice. ACS Chem. Neurosci. 11, 4316–4328. 10.1021/acschemneuro.0c00574 33216529

[B31] Liu G. Jiao Y. Lin Y. Hao H. Dou Y. Yang J. (2020). Discovery and Biological Evaluation of New Selective Acetylcholinesterase Inhibitors with Anti-aβ Aggregation Activity through Molecular Docking-Based Virtual Screening. Chem. Pharm. Bull. 68, 161–166. 10.1248/cpb.c19-00927 31813907

[B32] Marucci G. Buccioni M. Ben D. D. Lambertucci C. Volpini R. Amenta F. (2021). Efficacy of Acetylcholinesterase Inhibitors in Alzheimer's Disease. Neuropharmacology 190, 108352. 10.1016/j.neuropharm.2020.108352 33035532

[B33] Mollazadeh M. Mohammadi-Khanaposhtani M. Zonouzi A. Nadri H. Najafi Z. Larijani B. (2019). New Benzyl Pyridinium Derivatives Bearing 2, 4-dioxochroman Moiety as Potent Agents for Treatment of Alzheimer’s Disease: Design, Synthesis, Biological Evaluation, and Docking Study. Bioorg. Chem. 87, 506–515. 10.1016/j.bioorg.2019.03.012 30928873

[B34] Murakami K. Yamaguchi T. Izuo N. Kume T. Hara H. Irie K. (2020). Synthetic and Biophysical Studies on the Toxic Conformer in Amyloid β with the E22Δ Mutation in Alzheimer Pathology. ACS Chem. Neurosci. 11, 3017–3024. 10.1021/acschemneuro.0c00331 32790274

[B35] Nachon F. Carletti E. Ronco C. Trovaslet M. Nicolet Y. Jean L. (2013). Crystal Structures of Human Cholinesterases in Complex with Huprine W and Tacrine: Elements of Specificity for Anti-alzheimer's Drugs Targeting Acetyl-And Butyryl-Cholinesterase. Biochem. J. 453, 393–399. 10.1042/BJ20130013 23679855

[B36] Nasr T. Bondock S. Youns M. (2014). Anticancer Activity of New Coumarin Substituted Hydrazide–Hydrazone Derivatives. Eur. J. Med. Chem. 76, 539–548. 10.1016/j.ejmech.2014.02.026 24607878

[B37] Patel D. V. Patel N. R. Kanhed A. M. Teli D. M. Patel K. B. Gandhi P. M. (2020). Further Studies on Triazinoindoles as Potential Novel Multitarget-Directed Anti-alzheimer’s Agents. ACS Chem. Neurosci. 11, 3557–3574. 10.1021/acschemneuro.0c00448 33073564

[B38] Pérez-Cruz K. Moncada-Basualto M. Morales-Valenzuela J. Barriga-González G. Navarrete-Encina P. Núñez-Vergara L. (2018). Synthesis and Antioxidant Study of New Polyphenolic Hybrid-Coumarins. Arabian J. Chem. 11, 525–537. 10.1016/j.arabjc.2017.05.007

[B39] Pradhan K. Das G. Mondal P. Khan J. Barman S. Ghosh S. (2018). Genesis of Neuroprotective Peptoid from Aβ30–34 Inhibits Aβ Aggregation and AChE Activity. ACS Chem. Neurosci. 9, 2929–2940. 10.1021/acschemneuro.8b00071 30036464

[B40] Reddy E. K. Remya C. Mantosh K. Sajith A. M. Omkumar R. V. Sadasivan C. (2017). Novel Tacrine Derivatives Exhibiting Improved Acetylcholinesterase Inhibition: Design, Synthesis and Biological Evaluation. Eur. J. Med. Chem. 139, 367–377. 10.1016/j.ejmech.2017.08.013 28810188

[B41] Sang Z. Wang K. Han X. Cao M. Tan Z. Liu W. (2018). Design, Synthesis, and Evaluation of Novel Ferulic Acid Derivatives as Multi-Target-Directed Ligands for the Treatment of Alzheimer’s Disease. ACS Chem. Neurosci. 10, 1008–1024. 10.1021/acschemneuro.8b00530 30537804

[B42] Selkoe D. J. Hardy J. (2016). The Amyloid Hypothesis of Alzheimer's Disease at 25 Years. EMBO Mol. Med. 8, 595–608. 10.15252/emmm.201606210 27025652 PMC4888851

[B43] Sharma P. Tripathi A. Tripathi P. N. Singh S. S. Singh S. P. Shrivastava S. K. (2019). Novel Molecular Hybrids of N-Benzylpiperidine and 1, 3, 4-oxadiazole as Multitargeted Therapeutics to Treat Alzheimer’s Disease. ACS Chem. Neurosci. 10, 4361–4384. 10.1021/acschemneuro.9b00430 31491074

[B44] Trambauer J. Fukumori A. Steiner H. (2020). Pathogenic Aβ Generation in Familial Alzheimer’s Disease: Novel Mechanistic Insights and Therapeutic Implications. Curr. Opin. Neurobiol. 61, 73–81. 10.1016/j.conb.2020.01.011 32105841

[B45] Vafadarnejad F. Mahdavi M. Karimpour-Razkenari E. Edraki N. Sameem B. Khanavi M. (2018). Design and Synthesis of Novel Coumarin-Pyridinium Hybrids: *In Vitro* Cholinesterase Inhibitory Activity. Bioorg. Chem. 77, 311–319. 10.1016/j.bioorg.2018.01.013 29421707

[B46] Vyas N. A. Singh S. B. Kumbhar A. S. Ranade D. S. Walke G. R. Kulkarni P. P. (2018). Acetylcholinesterase and Aβ Aggregation Inhibition by Heterometallic Ruthenium (II)–platinum (II) Polypyridyl Complexes. Inorg. Chem. 57, 7524–7535. 10.1021/acs.inorgchem.8b00091 29893118

[B47] Wang Z. Wang Y. Li W. Mao F. Sun Y. Huang L. (2014). Design, Synthesis, and Evaluation of Multitarget-Directed Selenium-Containing Clioquinol Derivatives for the Treatment of Alzheimer’s Disease. ACS Chem. Neurosci. 5, 952–962. 10.1021/cn500119g 25121395

[B48] Witaicenis A. Seito L. N. Da Silveira Chagas A. de Almeida L. D. Luchini A. C. Rodrigues-Orsi P. (2014). Antioxidant and Intestinal Anti-inflammatory Effects of Plant-Derived Coumarin Derivatives. Phytomedicine 21, 240–246. 10.1016/j.phymed.2013.09.001 24176844

[B49] Xuan Z. Gu X. Yan S. Xie Y. Zhou Y. Zhang H. (2021). Dimeric Tacrine (10)-Hupyridone Effectively Combats Alzheimer’s Disease as A Multi-Target-Directed Ligand. ACS Chem. Neurosci. 12, 2462–2477. 10.1021/acschemneuro.1c00182 34156230

[B50] Yang Z. Song Q. Cao Z. Yu G. Liu Z. Tan Z. (2020). Design, Synthesis and Evaluation of Flurbiprofen-Clioquinol Hybrids as Multitarget-Directed Ligands against Alzheimer’s Disease. Bioorg. Med. Chem. 28, 115374. 10.1016/j.bmc.2020.115374 32089390

[B51] Zhang C. Du Q.-Y. Chen L.-D. Wu W.-H. Liao S.-Y. Yu L.-H. (2016). Design, Synthesis and Evaluation of Novel Tacrine-Multialkoxybenzene Hybrids as Multi-Targeted Compounds against Alzheimer's Disease. Eur. J. Med. Chem. 116, 200–209. 10.1016/j.ejmech.2016.03.077 27061983

[B52] Zou C. Montagna E. Shi Y. Peters F. Blazquez-Llorca L. Shi S. (2015). Intraneuronal APP and Extracellular Aβ Independently Cause Dendritic Spine Pathology in Transgenic Mouse Models of Alzheimer’s Disease. Acta Neuropathol. 129, 909–920. 10.1007/s00401-015-1421-4 25862638 PMC4436699

