# Supplementary material for: Novel Coumarin–Pyridine Hybrids as Potent Multi-Target Directed Ligands Aiming at Symptoms of Alzheimer’s Disease
Source: Front Chem. 2022 Jun 30;10:895483. doi: 10.3389/fchem.2022.895483 (PMC9280334; doi:10.3389/fchem.2022.895483)
Supplement: Supplementary file 1 [file DataSheet1.docx]

Novel Coumarin-Pyridine Hybrids as Potent Multi-target Directed Ligands Aiming at Symptoms of Alzheimer`s Disease

**Elaheh Babaei^1^, Leili Jalili-Baleh^2^, Hamid Nadri^3^, Tuba Tüylü Küçükkılınç^4^, Esin Öz^4^, Hamid Forootanfar^5^, Elaheh** **Hosseinzadeh^6^, Tayebeh** **Akbari^7^, Mehdi** **Shafiee Ardestani^8^, Loghman Firoozpour^2^,** [**Alireza Foroumadi**](https://pubmed.ncbi.nlm.nih.gov/?term=Foroumadi+A&cauthor_id=33687881)**^2^,** [**Mohammad Sharifzadeh**](https://pubmed.ncbi.nlm.nih.gov/?term=Sharifzadeh+M&cauthor_id=33687881)**^9^,** **Bi Bi Fatemeh Mirjalili^1^, Mehdi Khoobi^6, 8^***

^1^Department of Chemistry, Faculty of Science, Yazd University, Yazd, Iran

^2^Department of Medicinal Chemistry, Faculty of Pharmacy and Pharmaceutical Sciences, Tehran University of Medical Science, Tehran, Iran

^3^Faculty of Pharmacy, Shahid Sadoughi University of Medical Sciences, Yazd, Iran

^4^Hacettepe University, Faculty of Pharmacy, Department of Biochemistry, Ankara, Turkey

^5^Department of Pharmaceutical Biotechnology, Faculty of Pharmacy, Kerman University of Medical Sciences, Kerman, Iran

^6^The Institute of Pharmaceutical Sciences (TIPS), Tehran University of Medical Sciences, Tehran 1417614411, Iran

^7^Department of Microbiology, Islamic Azad University, North Tehran Branch, Tehran, Iran

^8^Department of Radiopharmacy, Faculty of Pharmacy, Tehran University of Medical Sciences, Tehran, Iran

^9^Department of Toxicology and Pharmacology, Faculty of Pharmacy, Tehran University of Medical Sciences, Tehran, Iran

*** Correspondence:**

Mehdi Khoobi,

The Institute of Pharmaceutical Sciences (TIPS), Tehran University of Medical Sciences, P.O. Box: 14155-6451, Tehran, Iran; Tel/Fax: +98-21-64121510; E-mails: m-khoobi@tums.ac.ir and mehdi.khoobi@gmail.com.


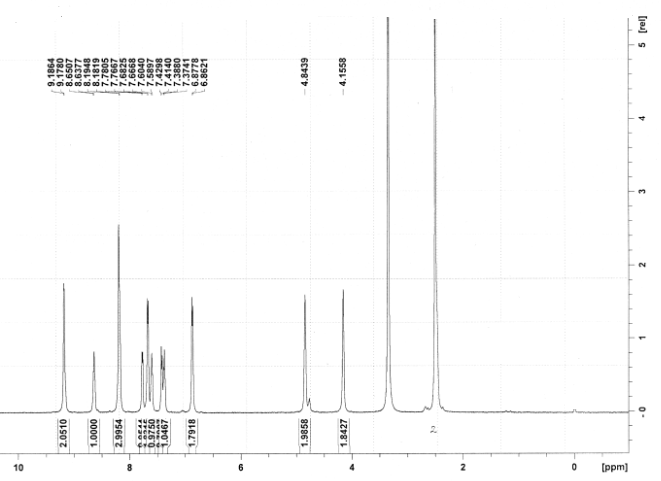


**3a**

^1^H NMR (400 MHz) spectrum of product (3a)


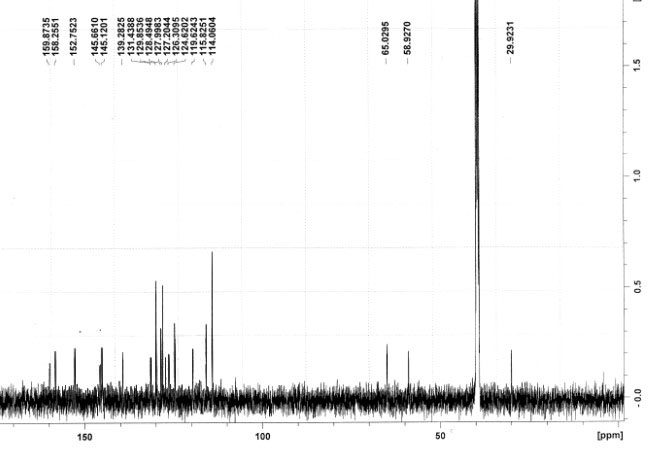

**(3a)**

The ^13^C NMR (100 MHz) spectrum of product (3a)

**
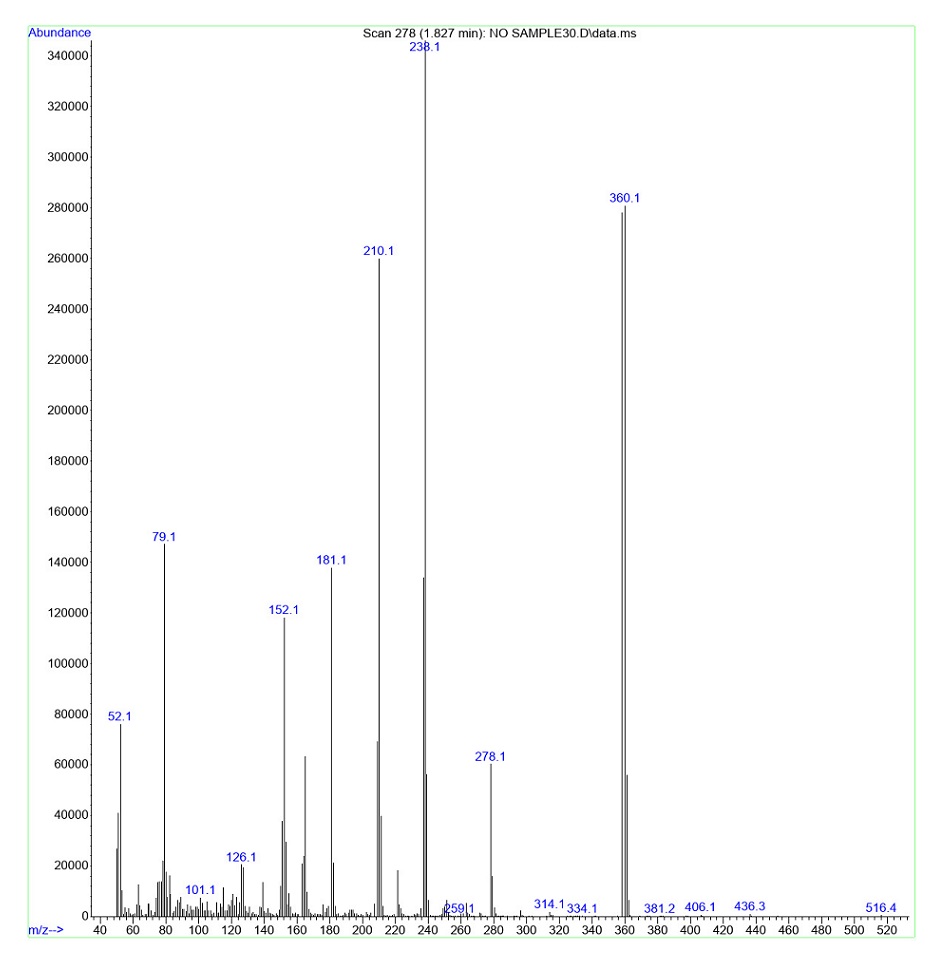
**

Mass spectrum of product (**3a**)


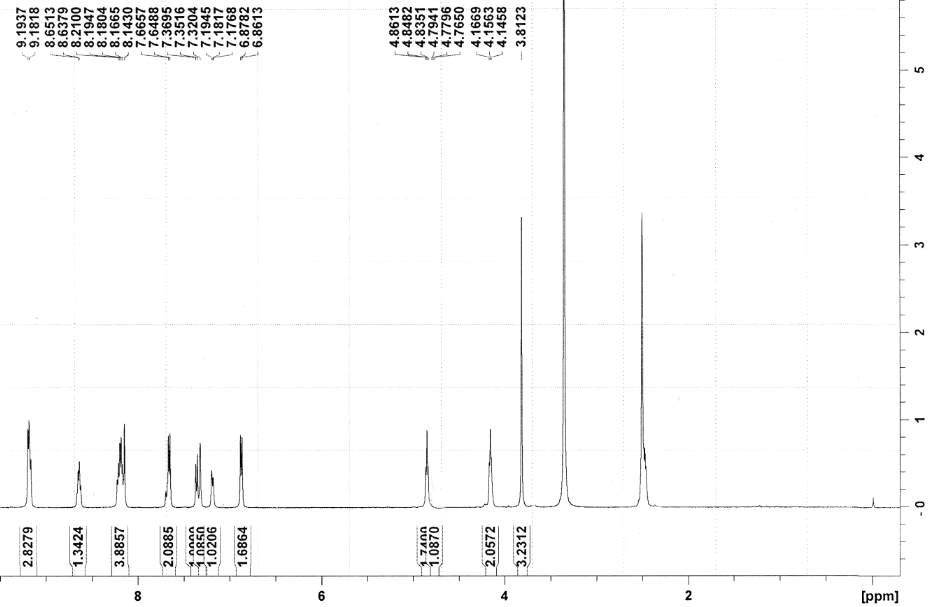


**(3b)**

^1^H NMR (400 MHz) spectrum of compound (3b)


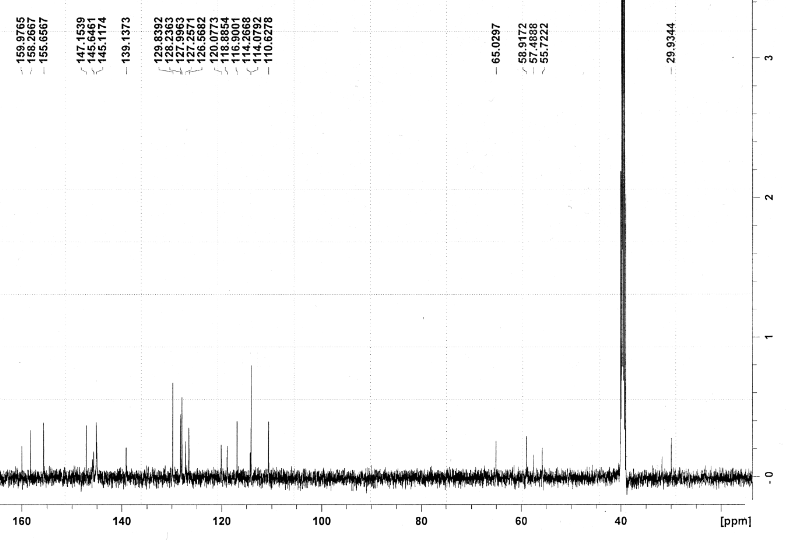


**(3b)**

^13^C NMR (100 MHz) spectrum of compound (3b)


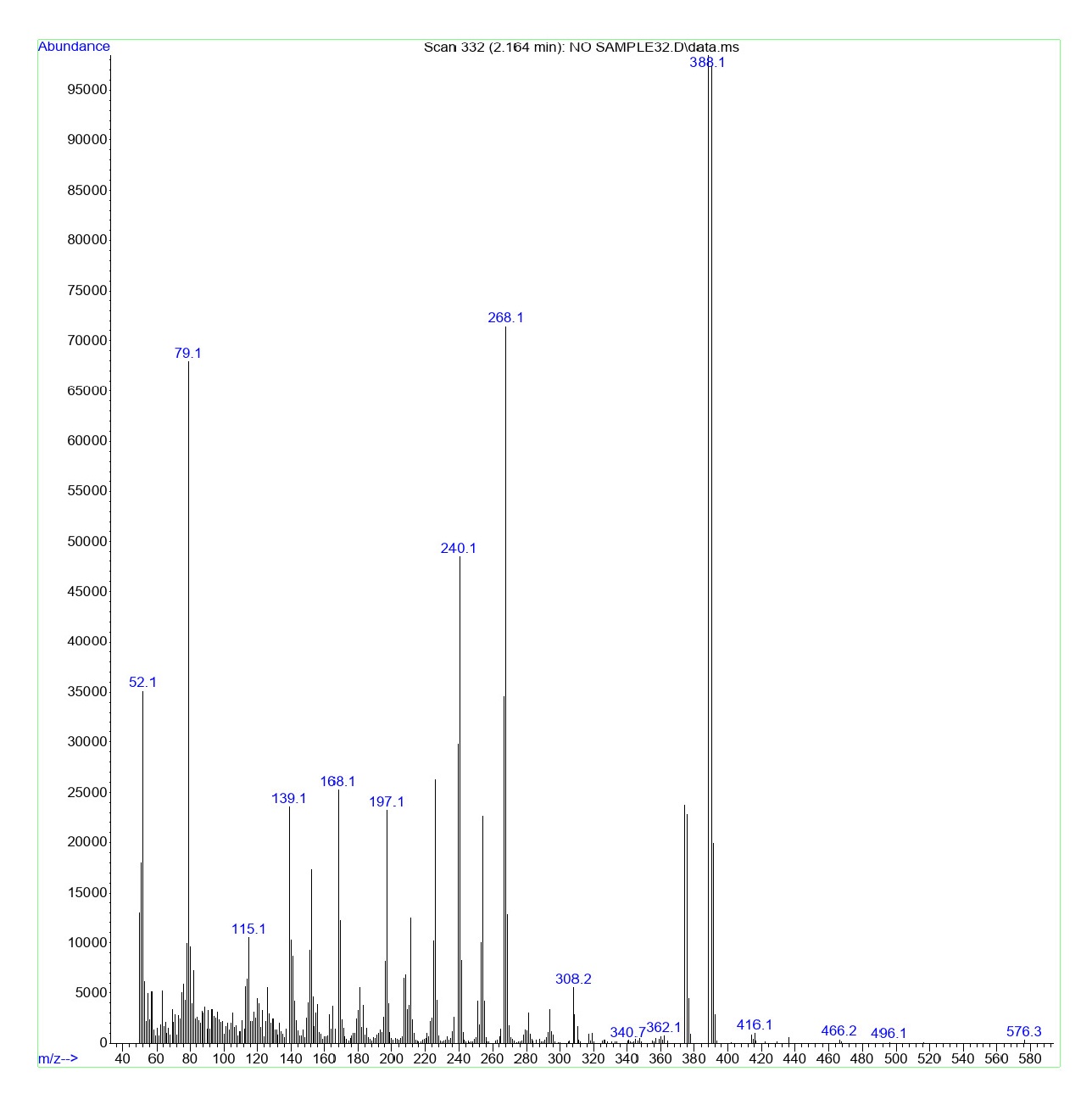


Mass spectrum of product (3b)


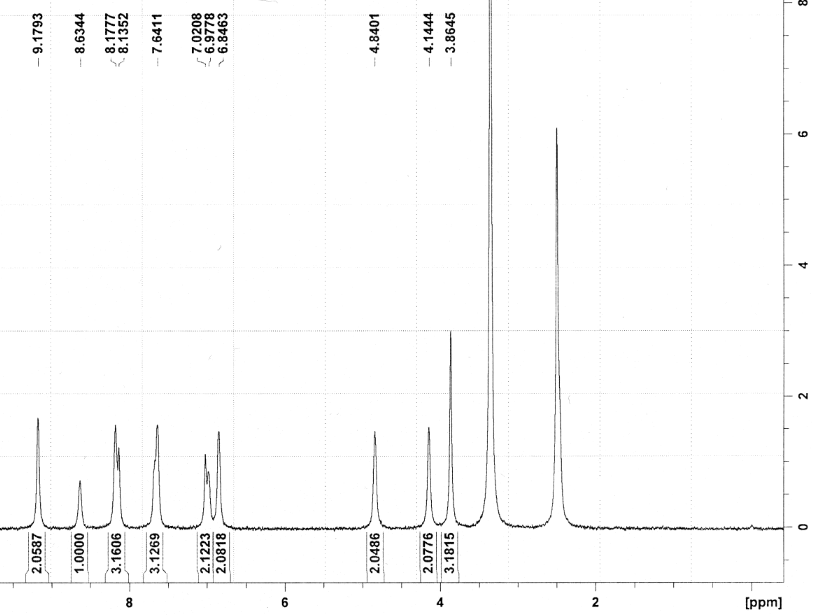


**(3c)**

The ^1^H NMR (400 MHz) spectrum of product (3c)


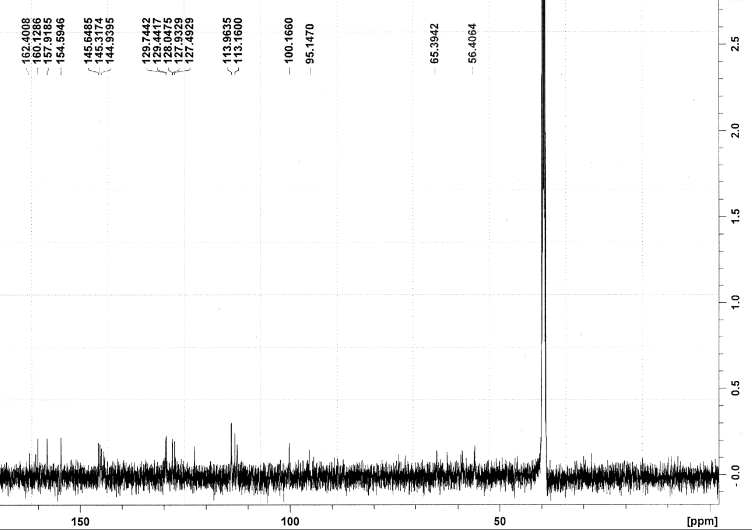


**(3c)**

The ^13^C NMR (100 MHz) spectrum of product (3c)


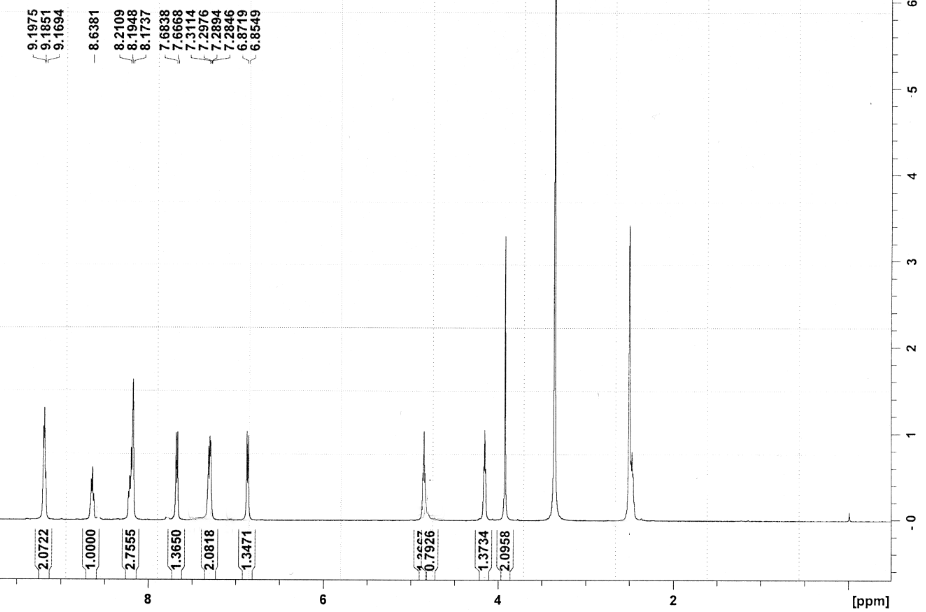


**(3d)**

The ^1^H NMR (400 MHz) spectrum of product (3d)


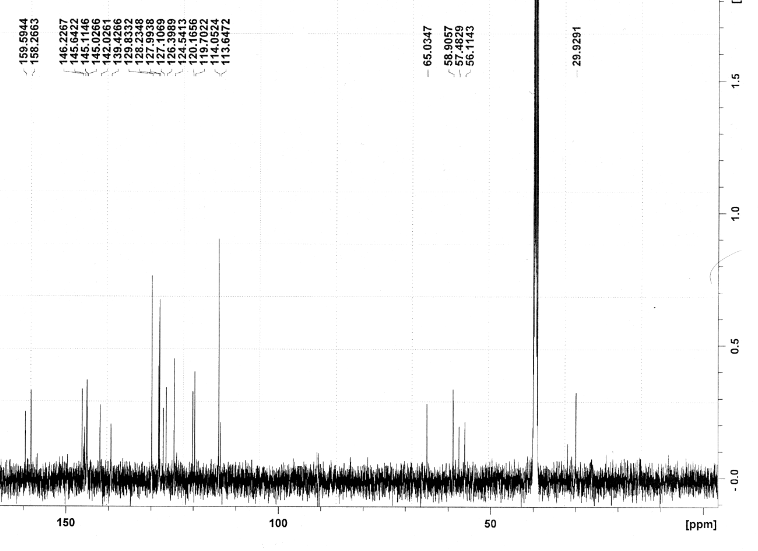


**(3d))**

The ^13^C NMR (100 MHz) spectrum of product (3d)


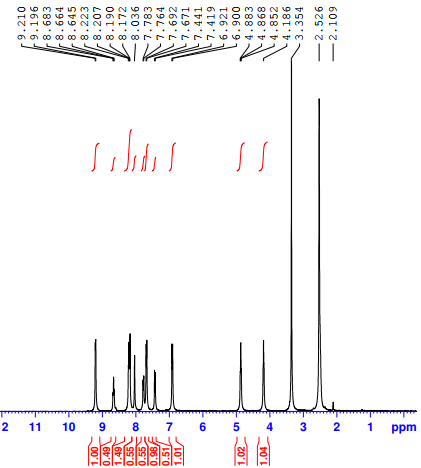

**(3e)**

The ^1^H NMR (400 MHz) spectrum of product (3e)


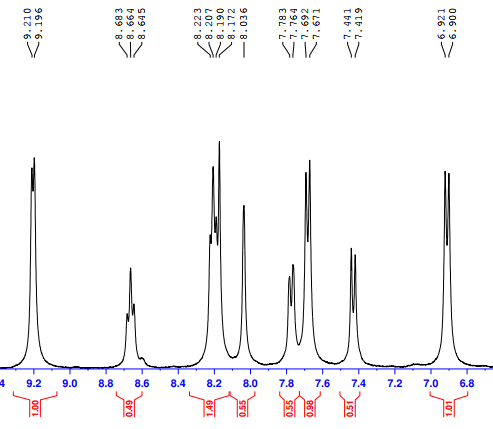

**(3e)**

The ^1^H NMR (400 MHz) spectrum of product (3e)


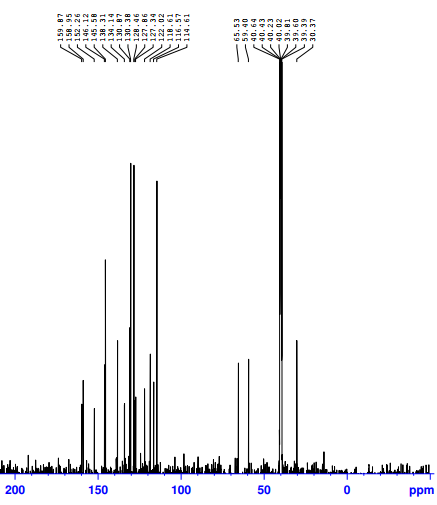


**(3e)**

The ^13^C NMR (100 MHz) spectrum of product (3e)


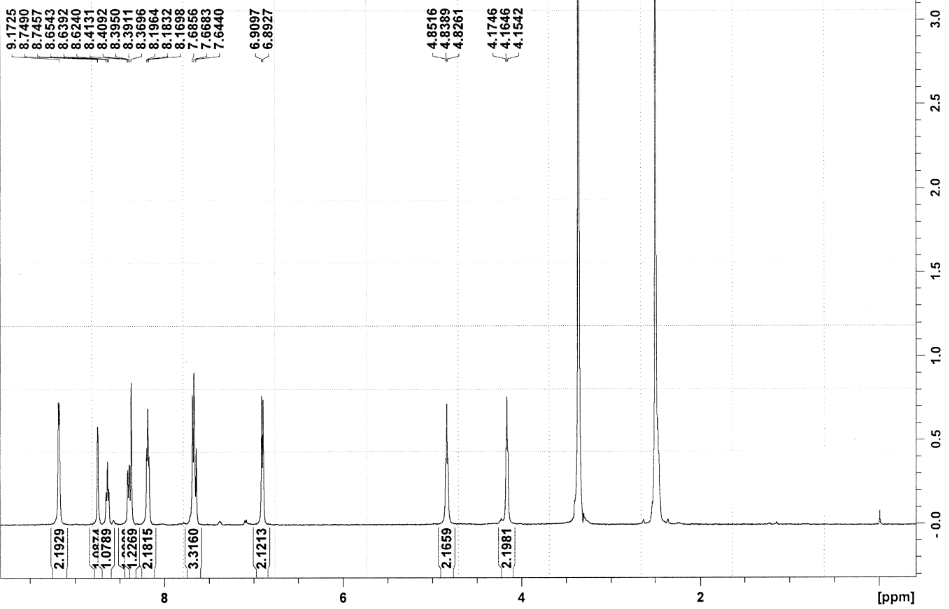


**(3f)**

The ^1^H NMR (400 MHz) spectrum of product (3f)


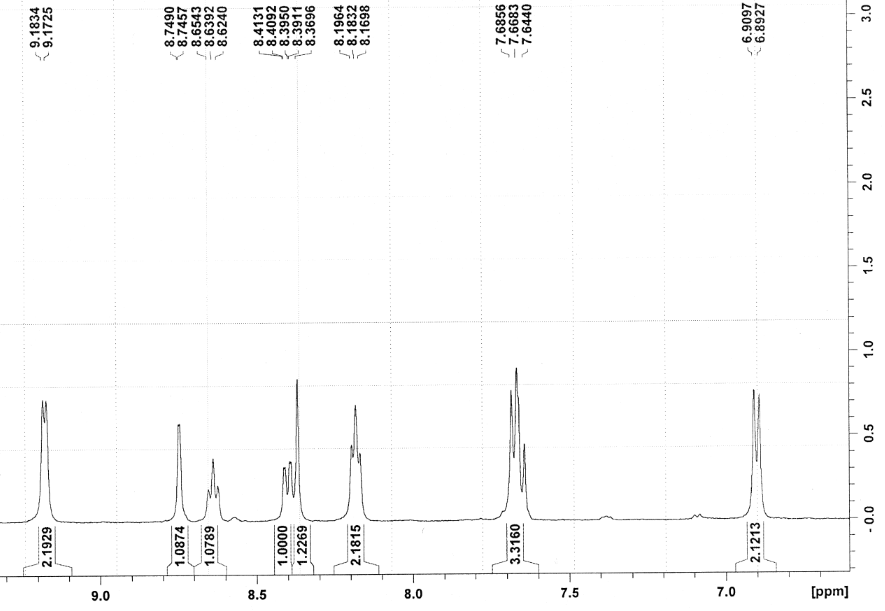


**(3f)**

The ^1^H NMR (400 MHz) spectrum of product (3f)


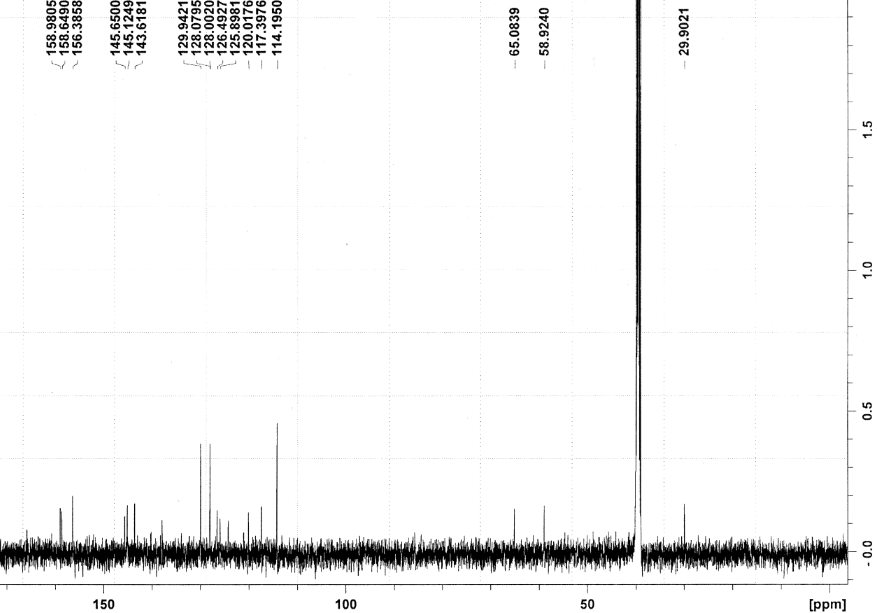


**(3f)**

The ^13^C NMR (100 MHz) spectrum of product (3f)

**
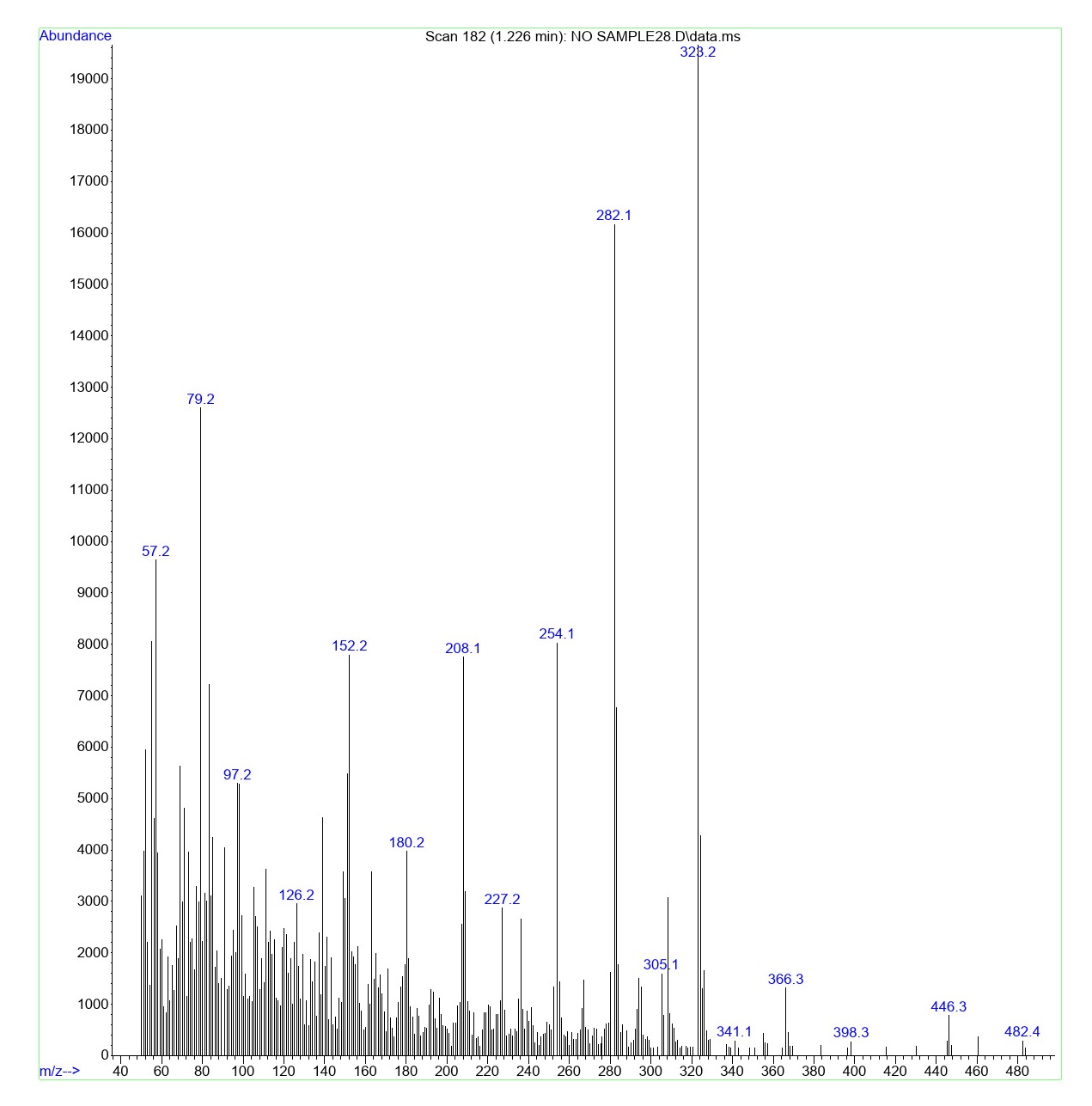
**

Mass spectrum of product (3f)


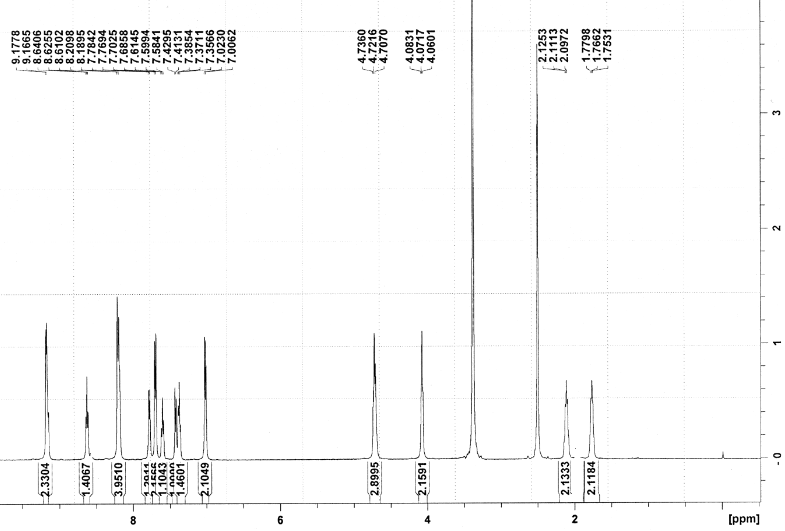


**(3g)**

The ^1^H NMR (400 MHz) spectrum of product (3g)


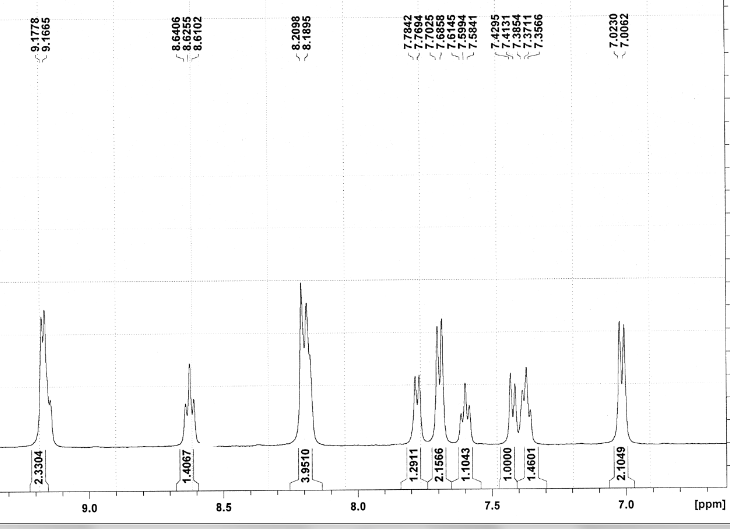


**(3g)**

The ^1^H NMR (400 MHz) spectrum of product (3g)


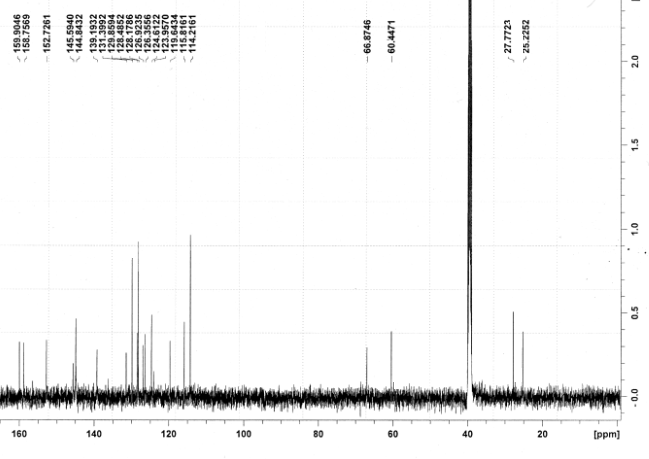


**(3g)**

The ^13^C NMR (100 MHz) spectrum of product (3g)

**
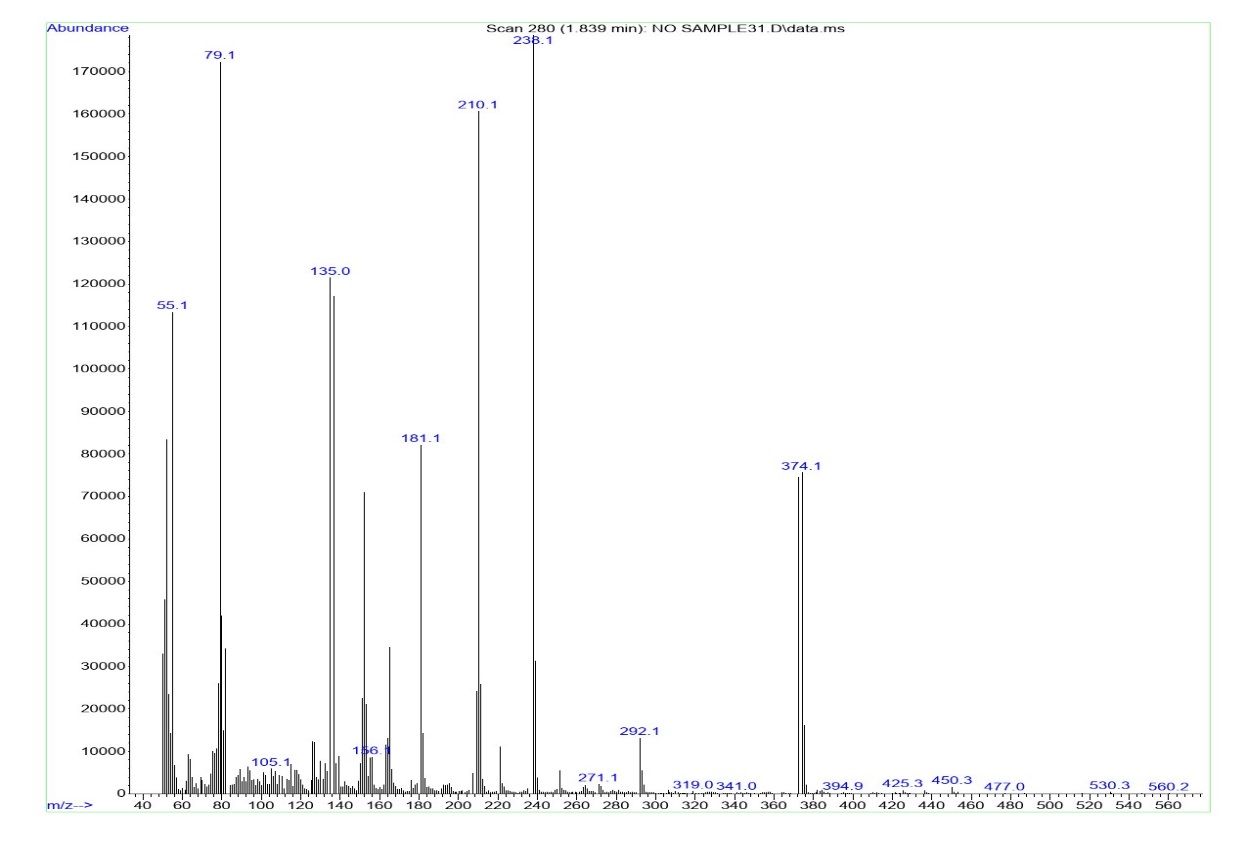
**

Mass spectrum of product (3g)


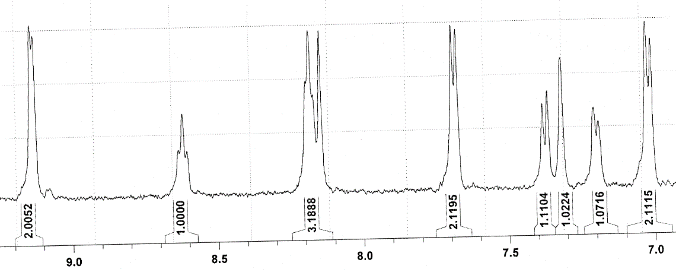

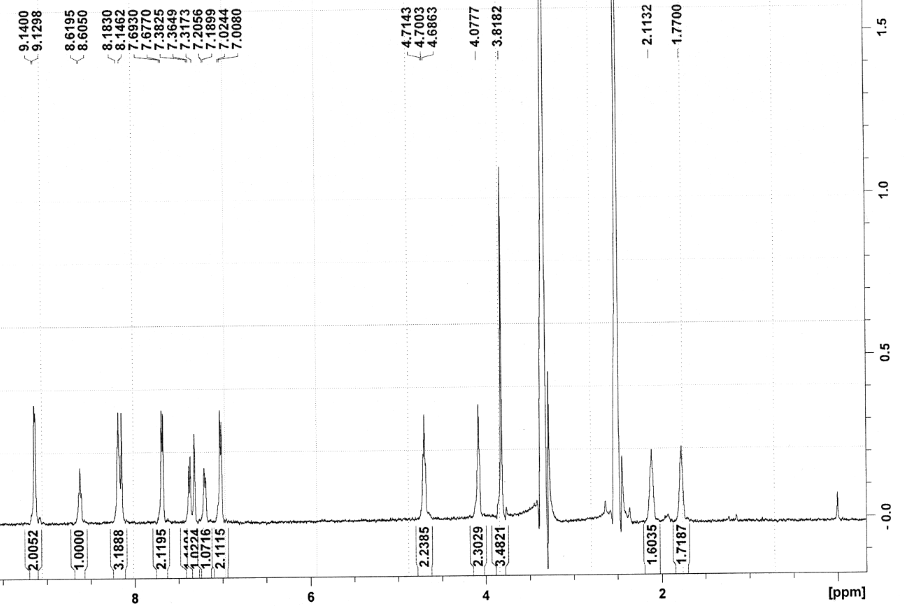


**(3h)**

The ^1^H NMR (400 MHz) spectrum of product (3h)


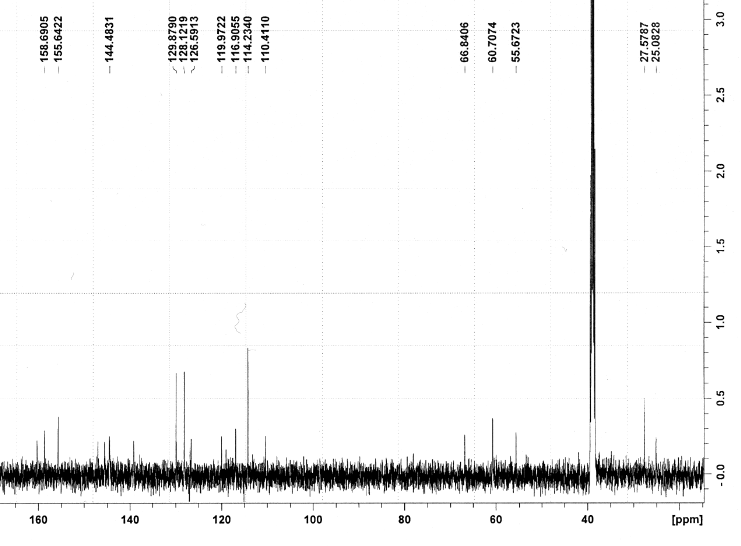


**(3h)**

The ^13^C NMR (100 MHz) spectrum of product (3h)


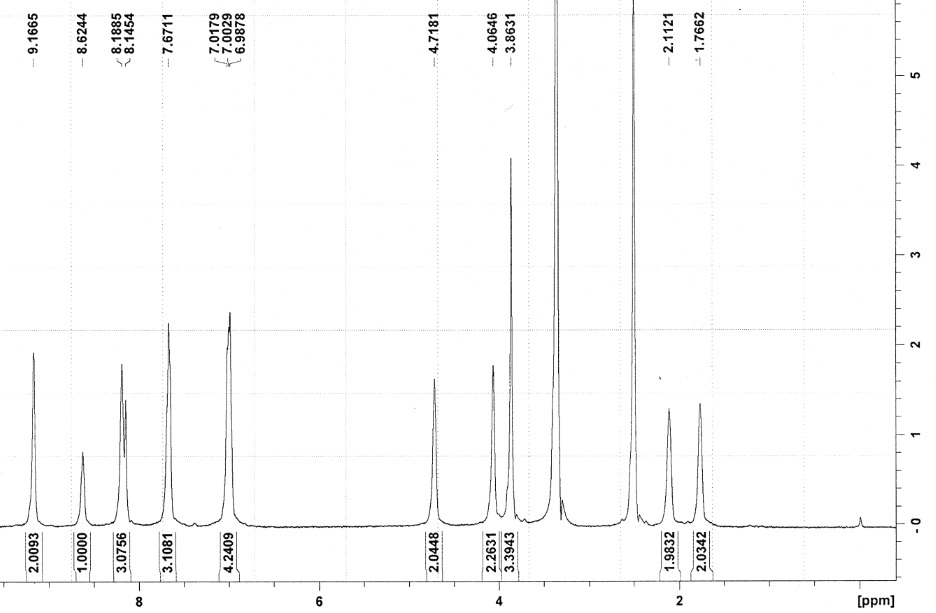


**(3i)**

The ^1^H NMR (400 MHz) spectrum of product (3i)


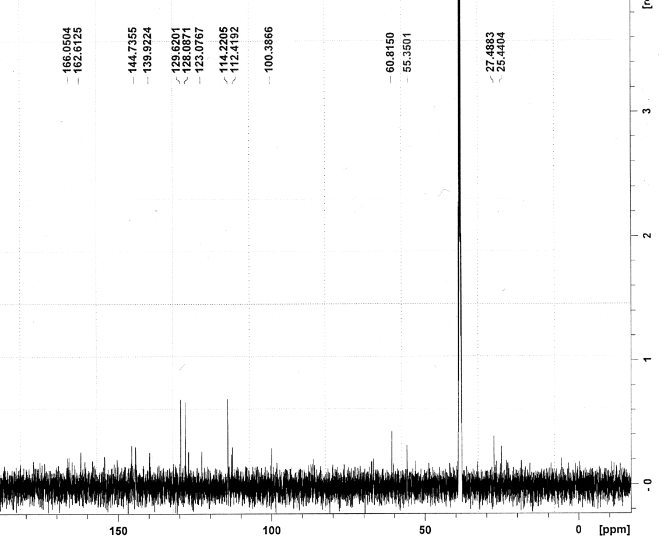


**(3i)**

The ^13^C NMR (100 MHz) spectrum of product (3i)


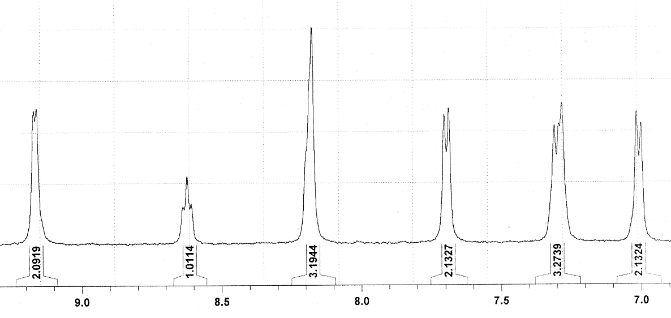

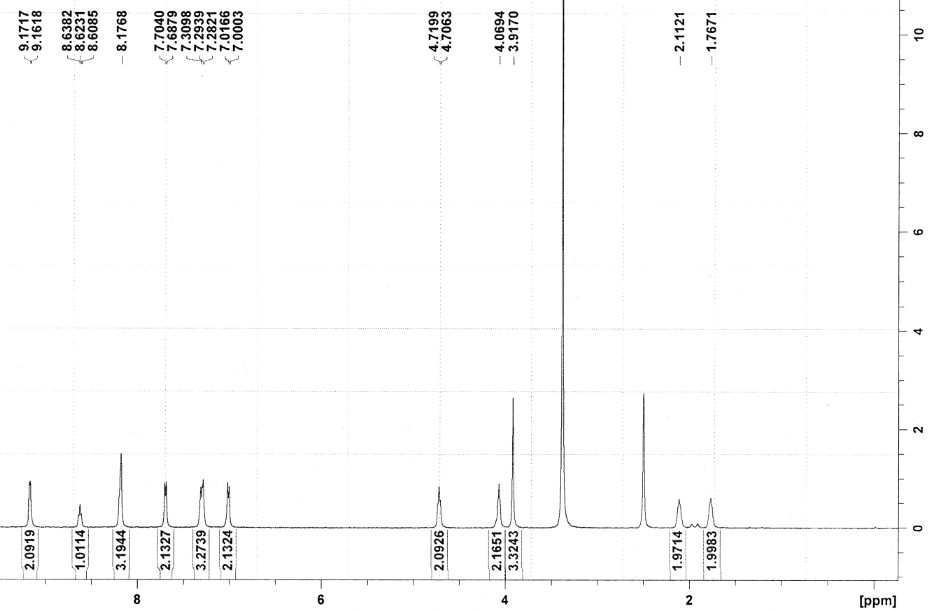


**(3j)**

The ^1^H NMR (400 MHz) spectrum of product (3j)


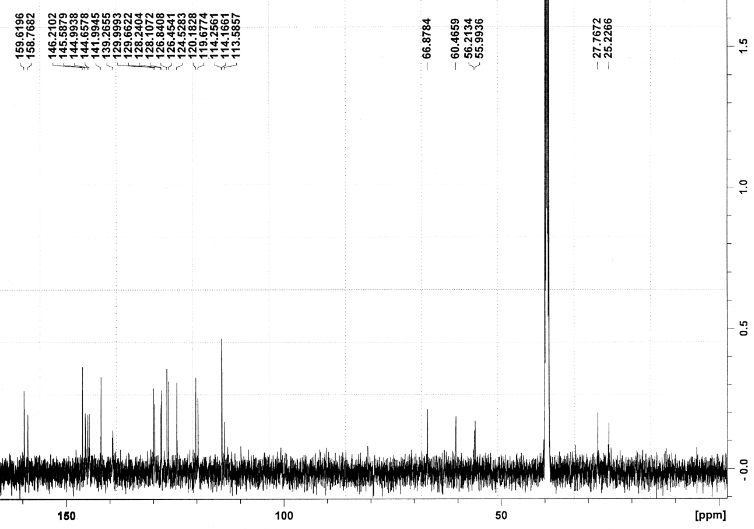


**(3j)**

The ^13^C NMR (100 MHz) spectrum of product (3j)


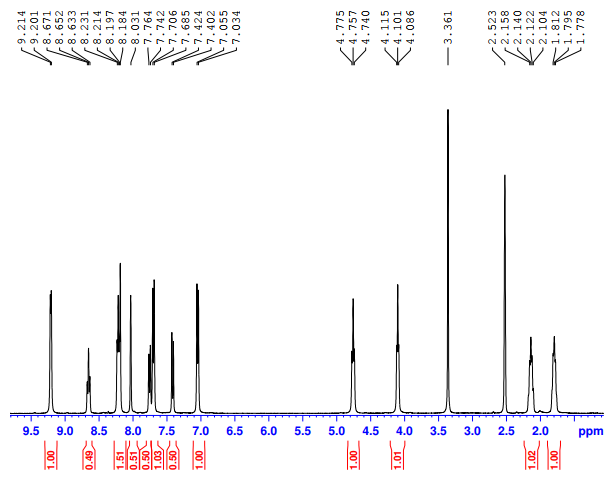


**(3k)**

The ^1^H NMR (400 MHz) spectrum of product (3k)


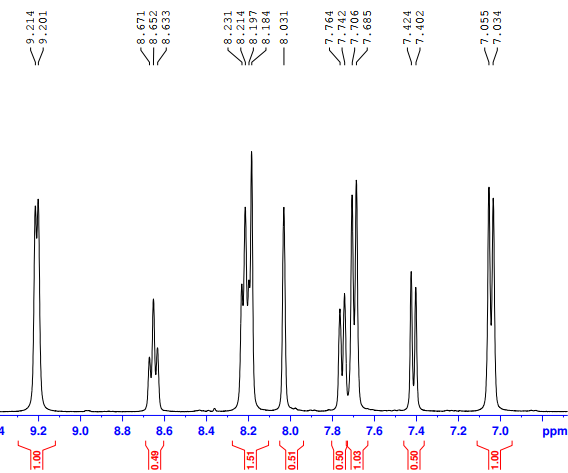


**(3k)**

The ^1^H NMR (400 MHz) spectrum of product (3k)


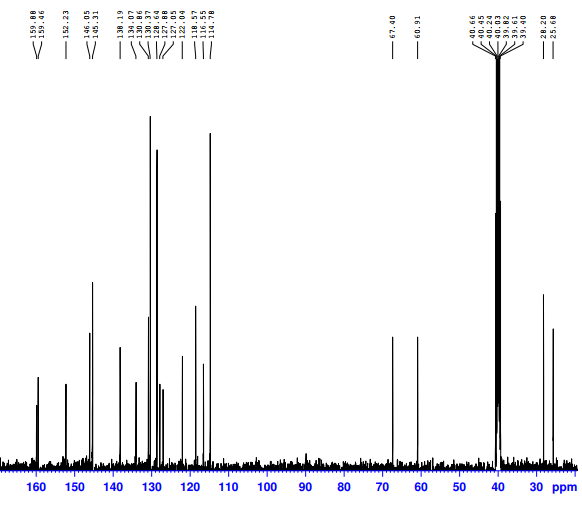


**(3k)**

The ^13^C NMR (100 MHz) spectrum of product (3k)


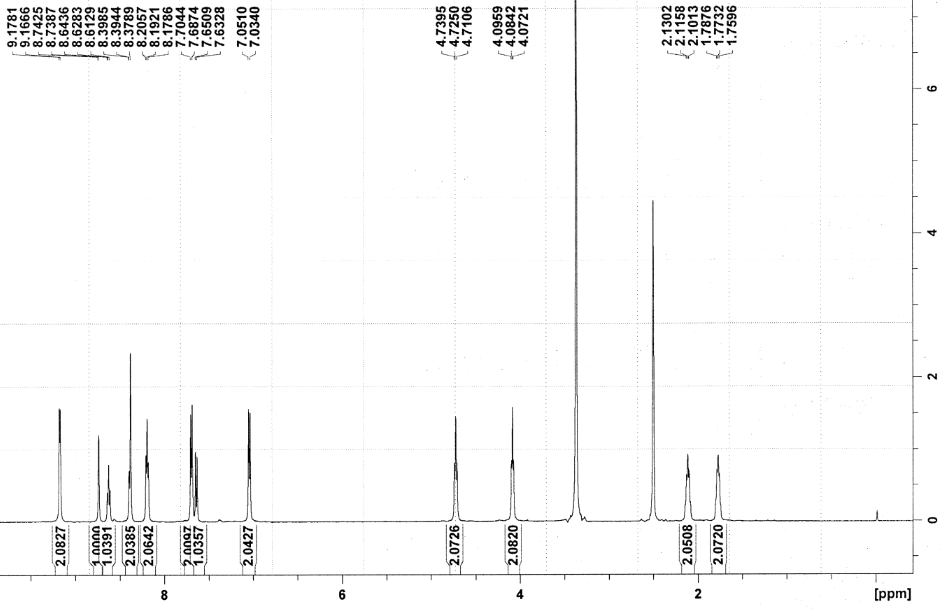


**(3l)**

The ^1^H NMR (400 MHz) spectrum of product (3l)


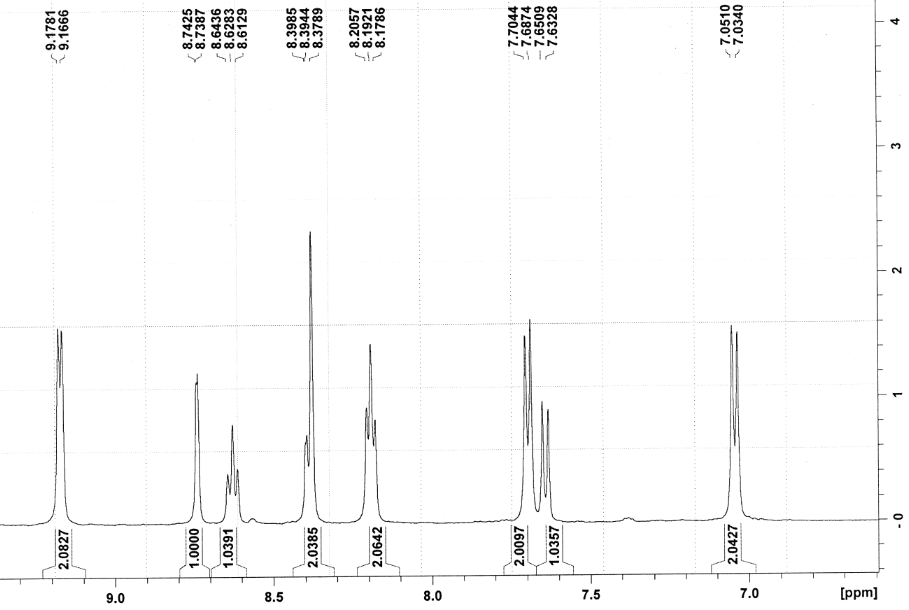


**(3l)**

The ^1^H NMR (400 MHz) spectrum of product (3l)


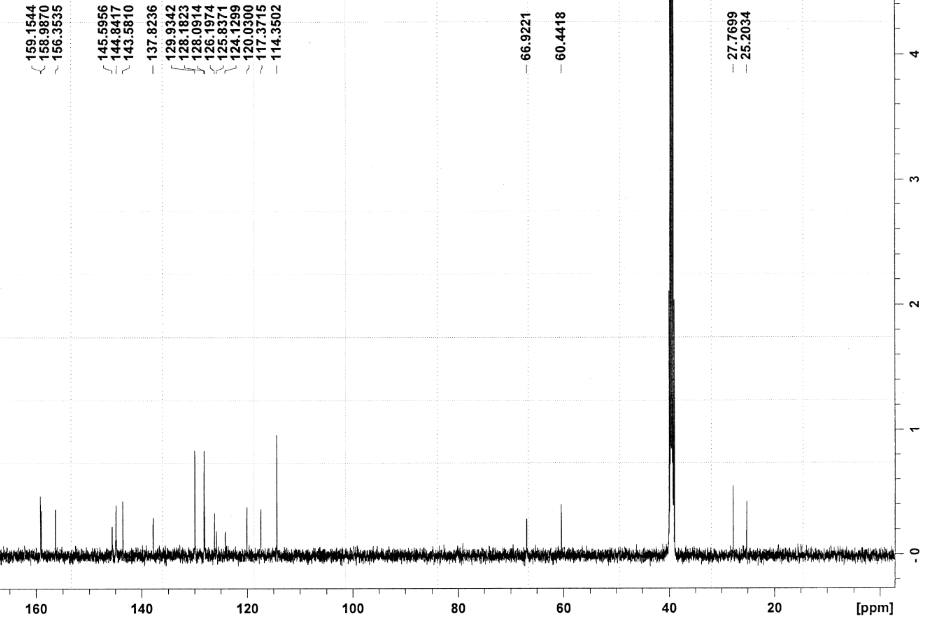


**(3l)**

The ^13^C NMR (100 MHz) spectrum of product (3l)

**
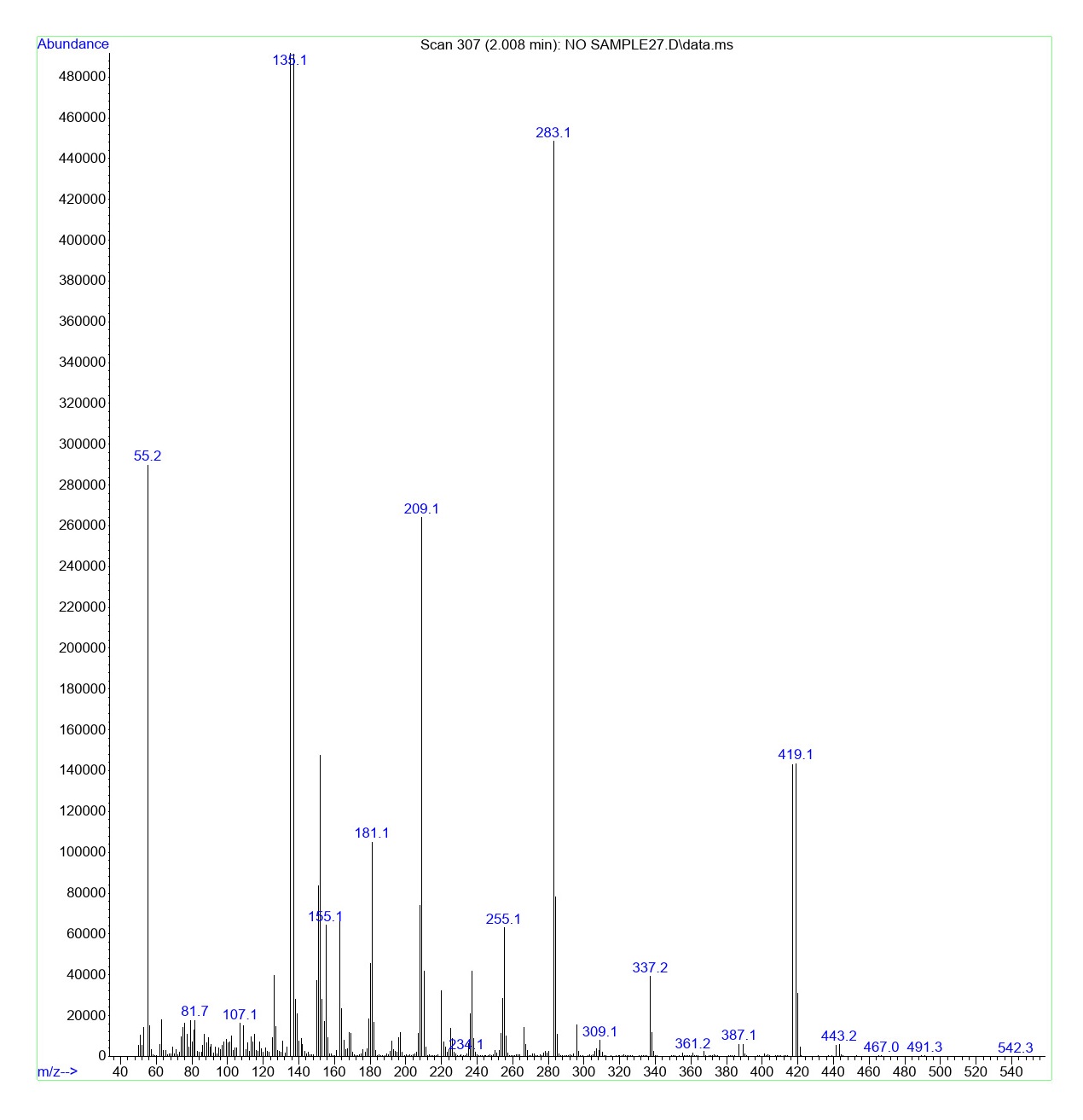
**

Mass spectrum of product (**3l**)


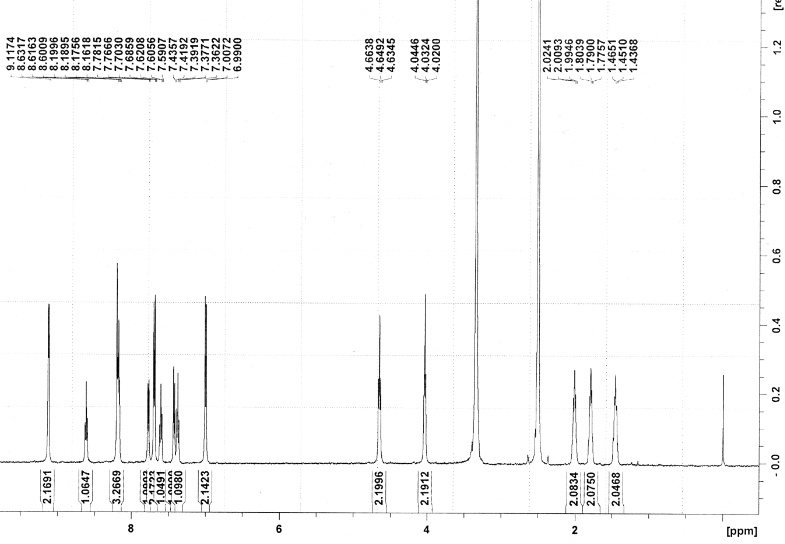


**(3m)**

he ^1^H NMR (400 MHz) spectrum of product (3m)
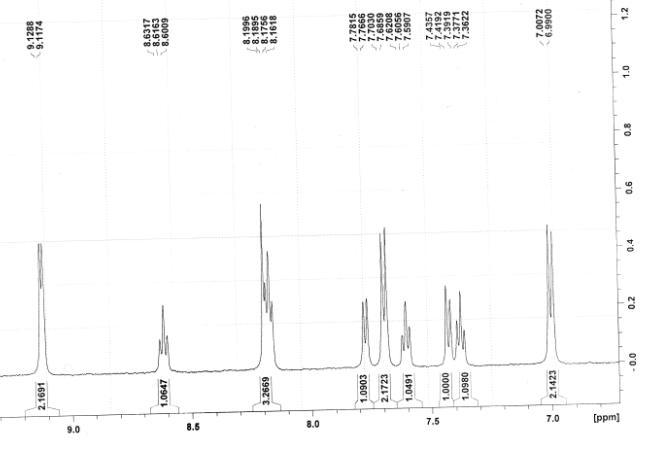

**(3m)**

The ^1^H NMR (400 MHz) spectrum of product (3m)


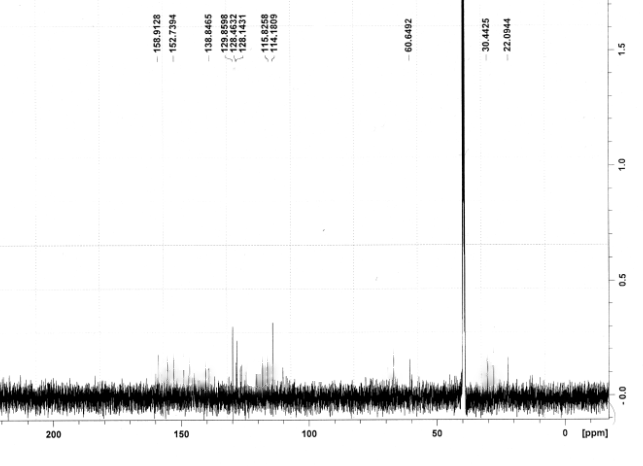


**(3m)**

The ^13^C NMR (100 MHz) spectrum of product (3m)


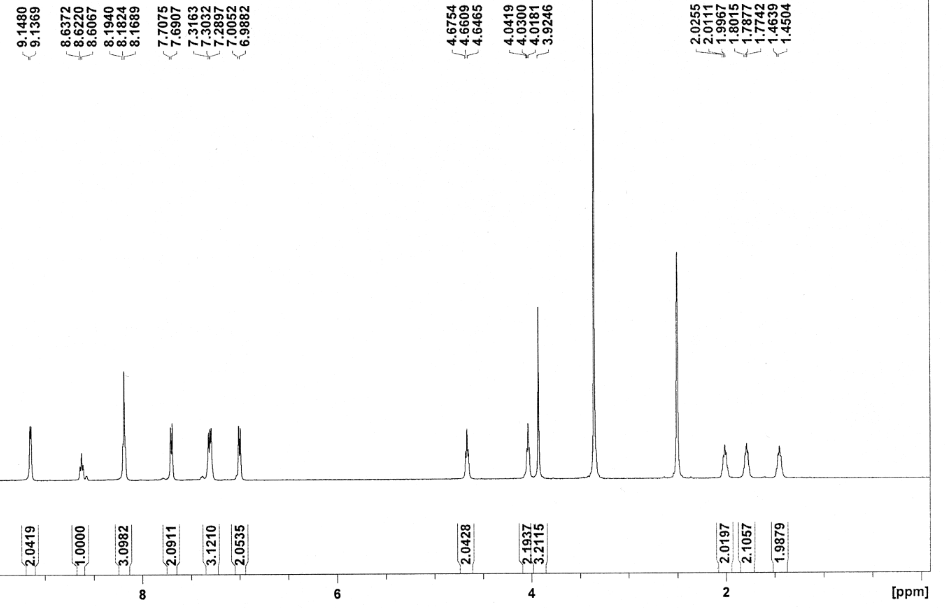


**(3n)**

The ^1^H NMR (400 MHz) spectrum of product (3n)


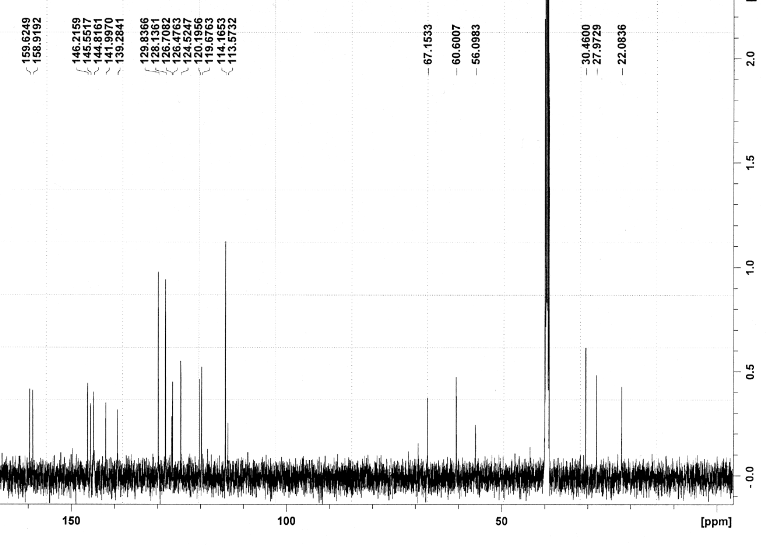


**(3n)**

The ^13^C NMR (100 MHz) spectrum of product (3n)


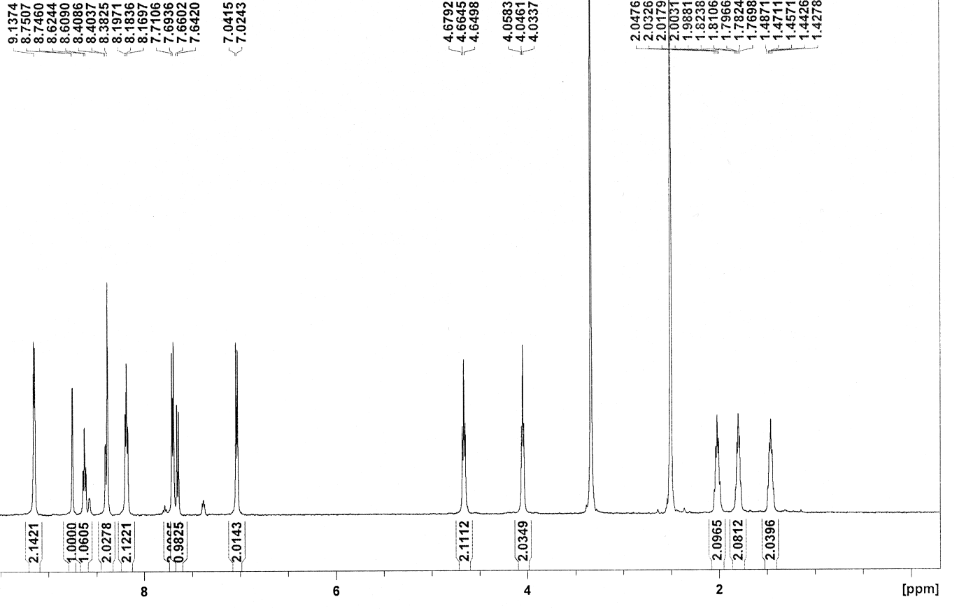


**(3o)**

The ^1^H NMR (400 MHz) spectrum of product (3o)


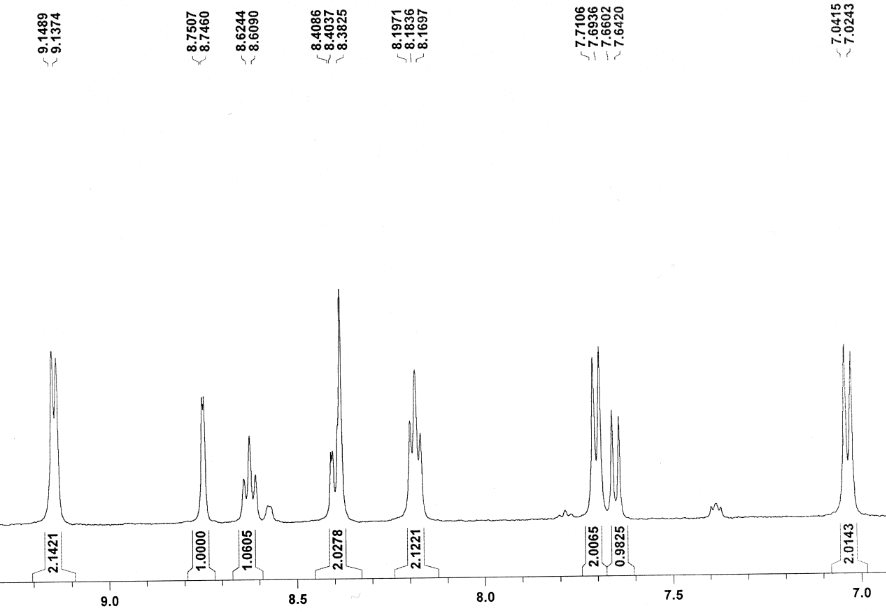


**(3o)**

The ^1^H NMR (400 MHz) spectrum of product (3o)


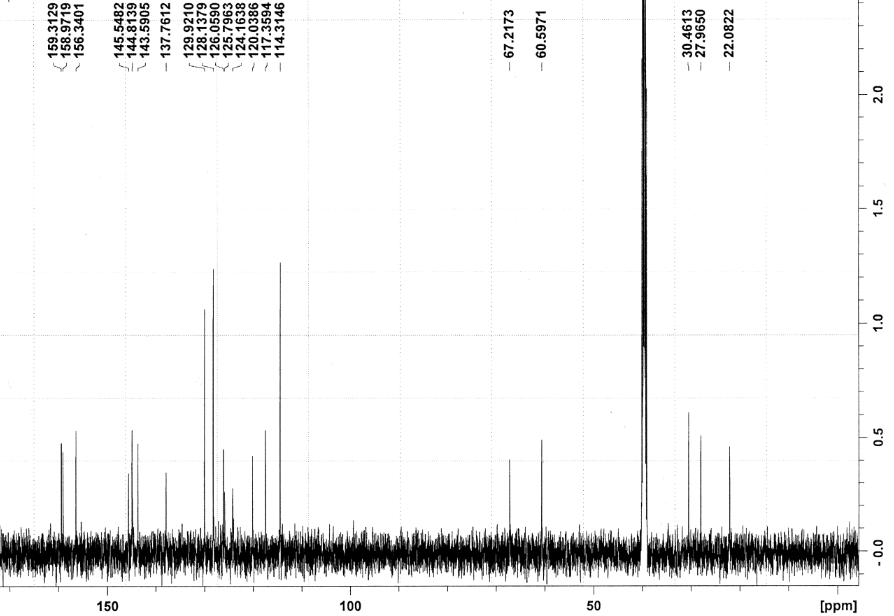


**(3o)**

The ^13^C NMR (100 MHz) spectrum of product (3o)


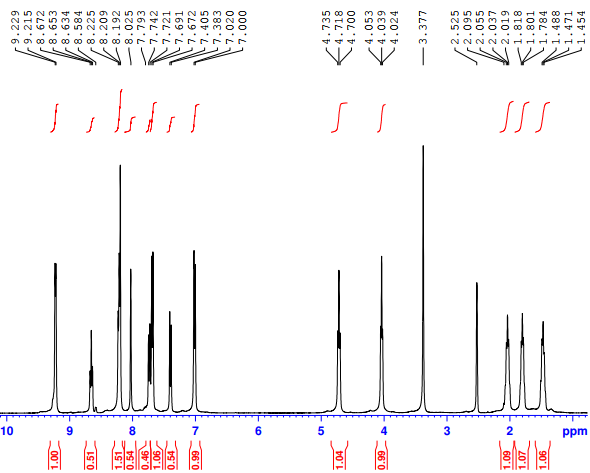

**(3p)**

The ^1^H NMR (400 MHz) spectrum of product (3p)


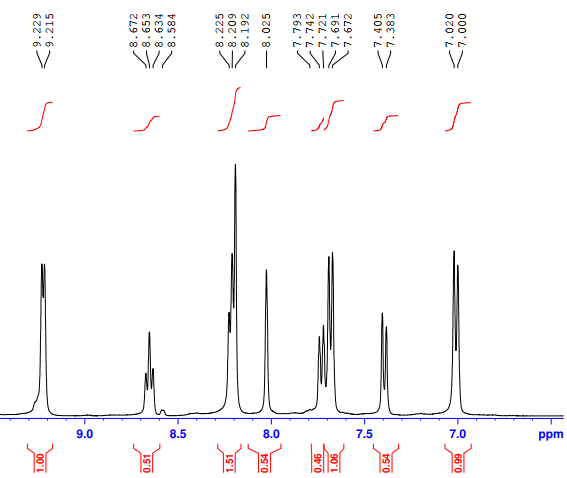


**(3p)**

The ^1^H NMR (400 MHz) spectrum of product (3p)


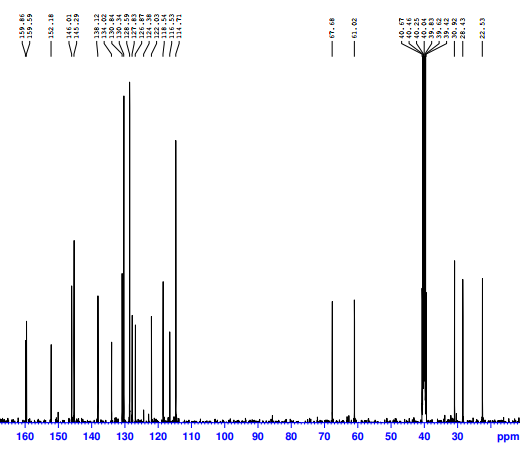


**(3p)**

The ^13^C NMR (100 MHz) spectrum of product (3p)


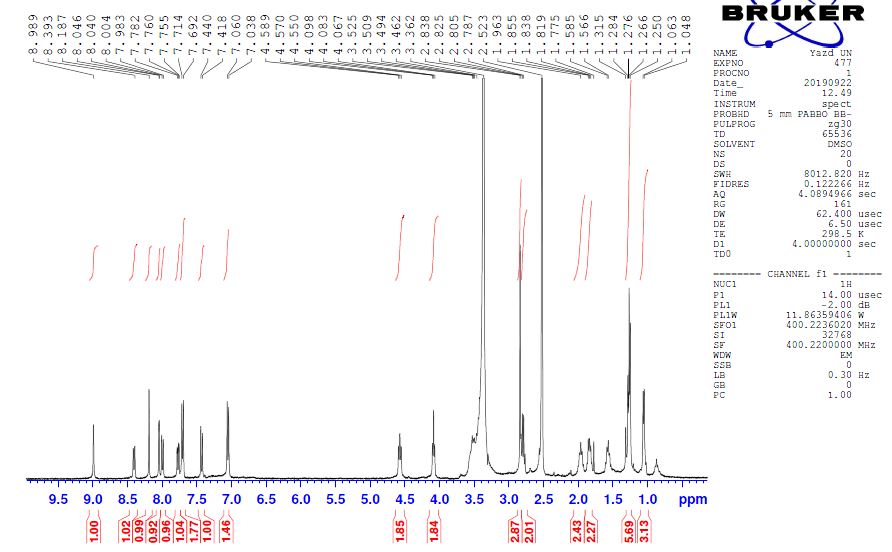


**(3q)**

The ^1^H NMR (400 MHz) spectrum of product (3q)


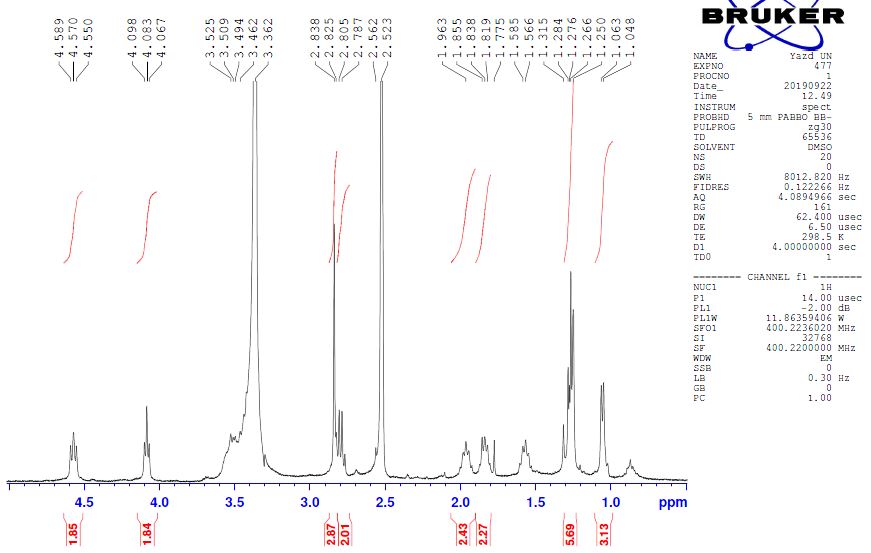


**(3q)**

The ^1^H NMR (400 MHz) spectrum of product (3q)


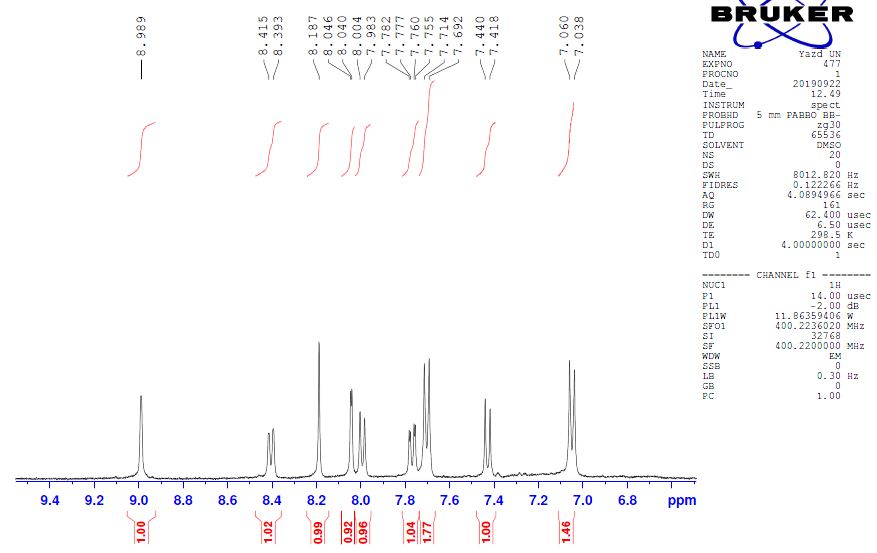


**(3q)**

The ^1^H NMR (400 MHz) spectrum of product (3q)


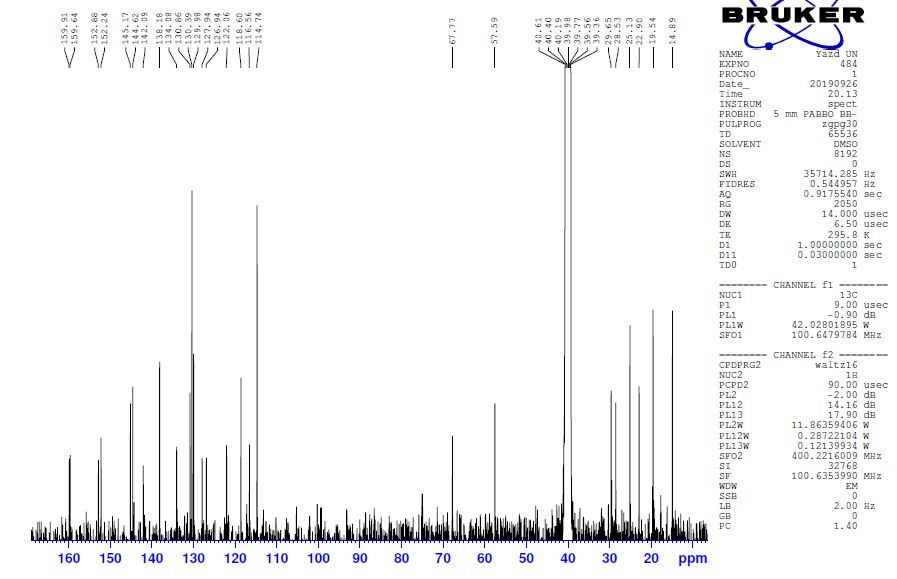


**(3q)**

The ^13^C NMR (100 MHz) spectrum of product (3q)


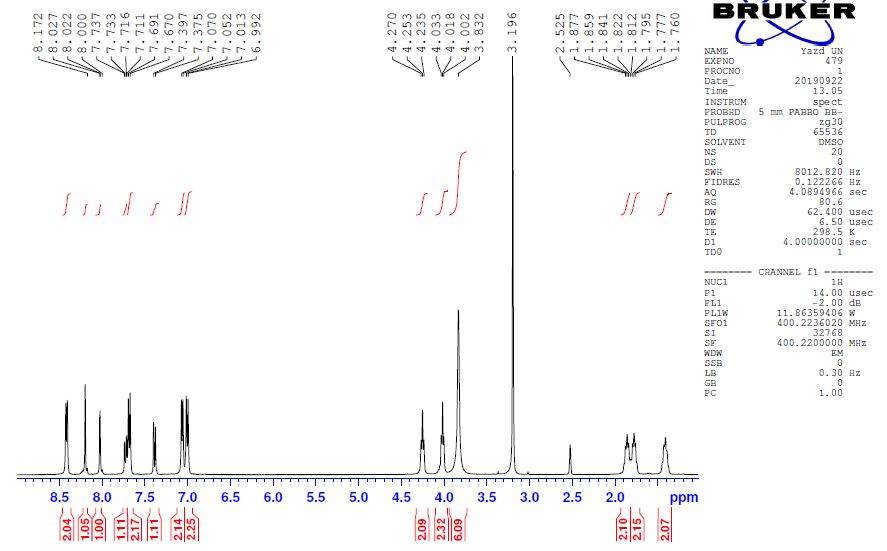


**(3r)**

The ^1^H NMR (400 MHz) spectrum of product (3r)

**
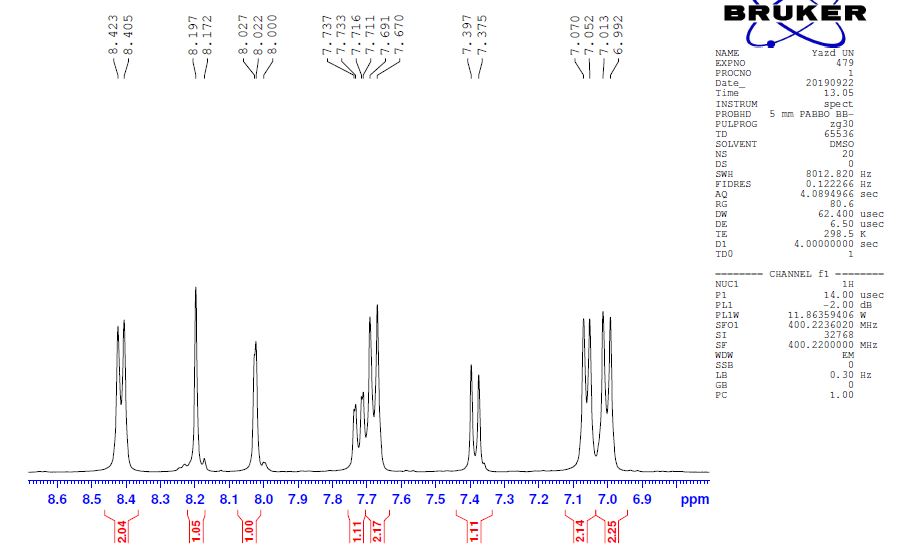
**

**(3r)**

The ^1^H NMR (400 MHz) spectrum of product (3r) *
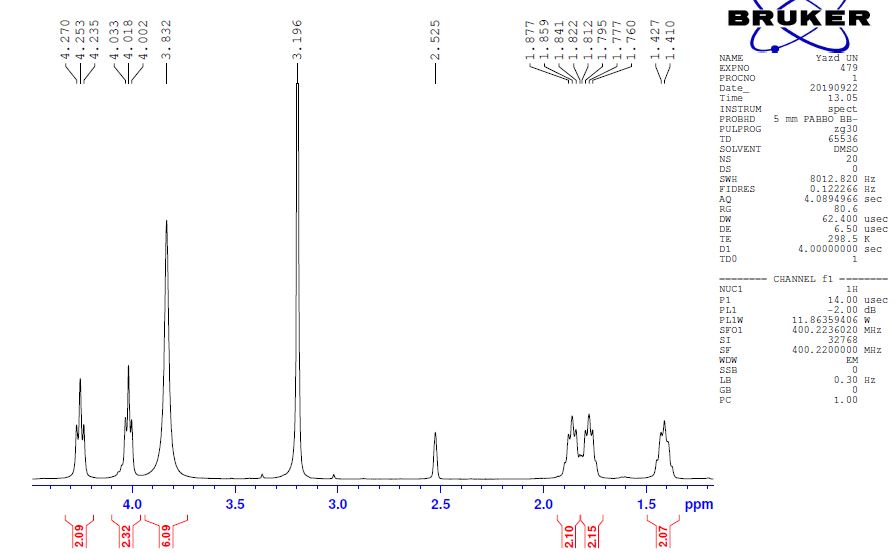
*

**(3r)**

The ^1^H NMR (400 MHz) spectrum of product (3r)


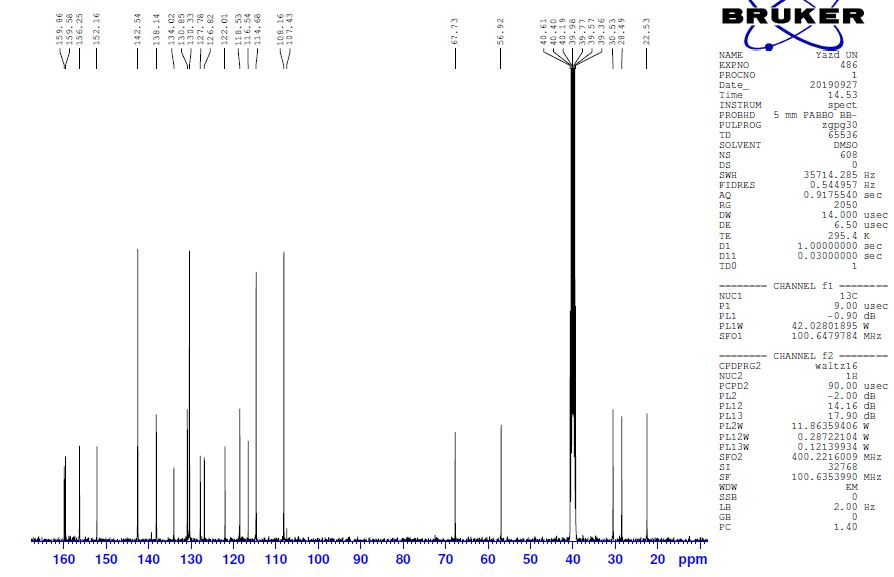


**(3r)**

The ^13^C NMR (100 MHz) spectrum of product (3r)


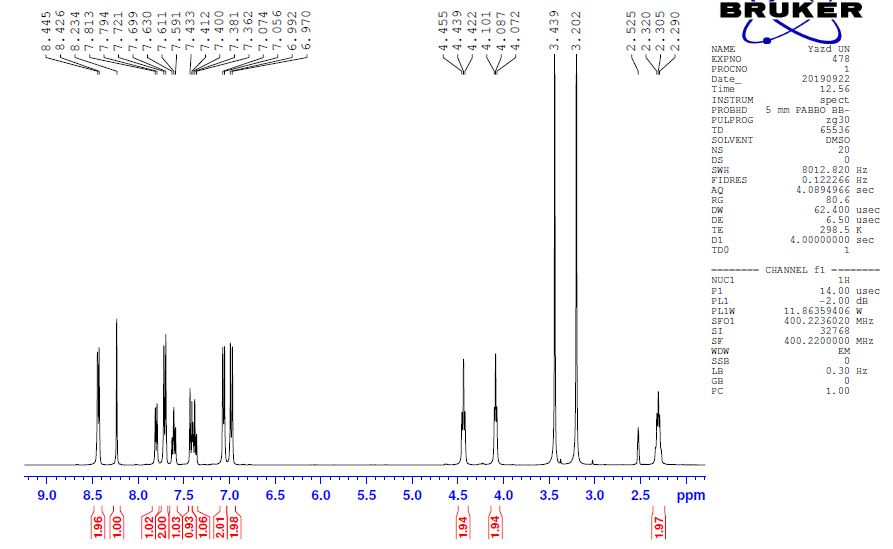


**(3s)**

The ^1^H NMR (400 MHz) spectrum of product (3s)


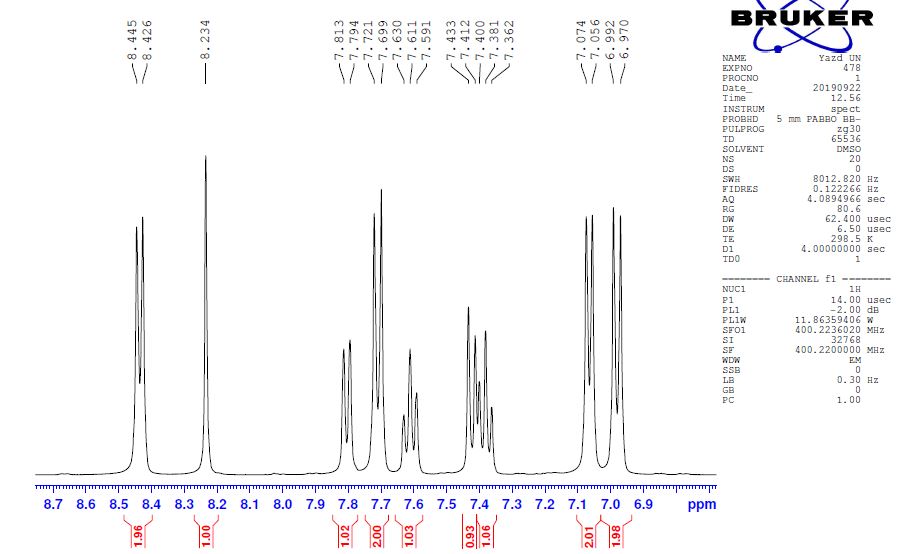


**(3s)**

The ^1^H NMR (400 MHz) spectrum of product (3s)

**
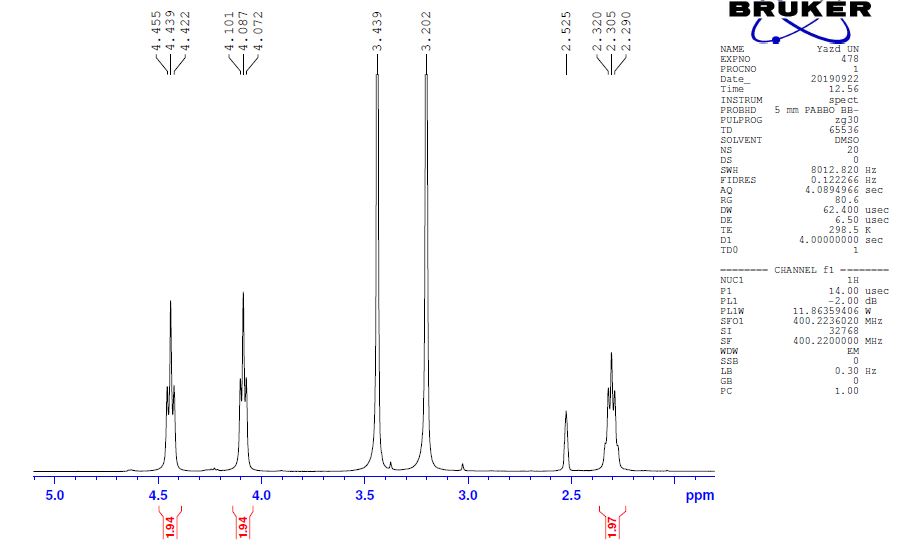
**

**(3s)**

The ^1^H NMR (400 MHz) spectrum of product (3s)


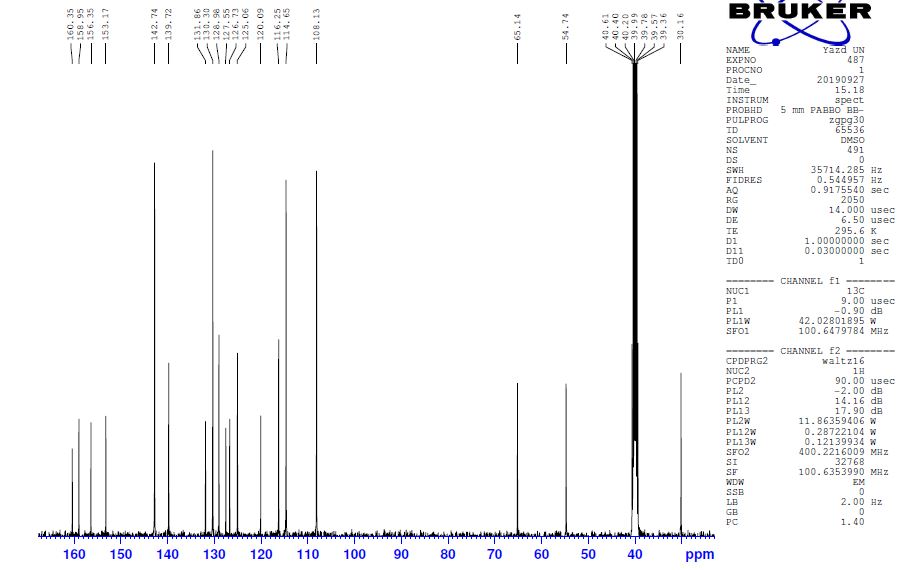


**(3s)**

The ^13^C NMR (100 MHz) spectrum of product (3s)


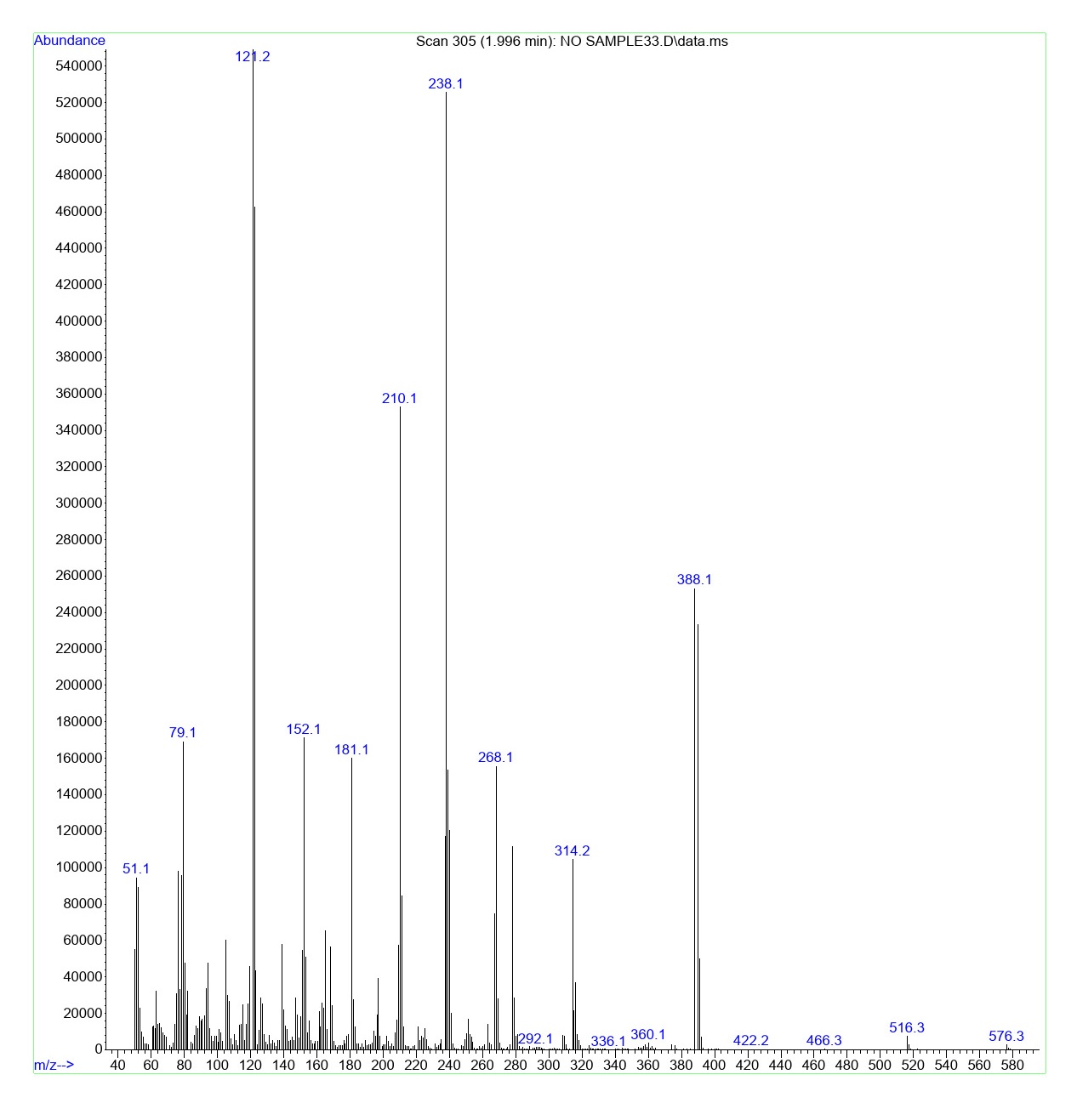


Mass spectrum of product (3s)

**
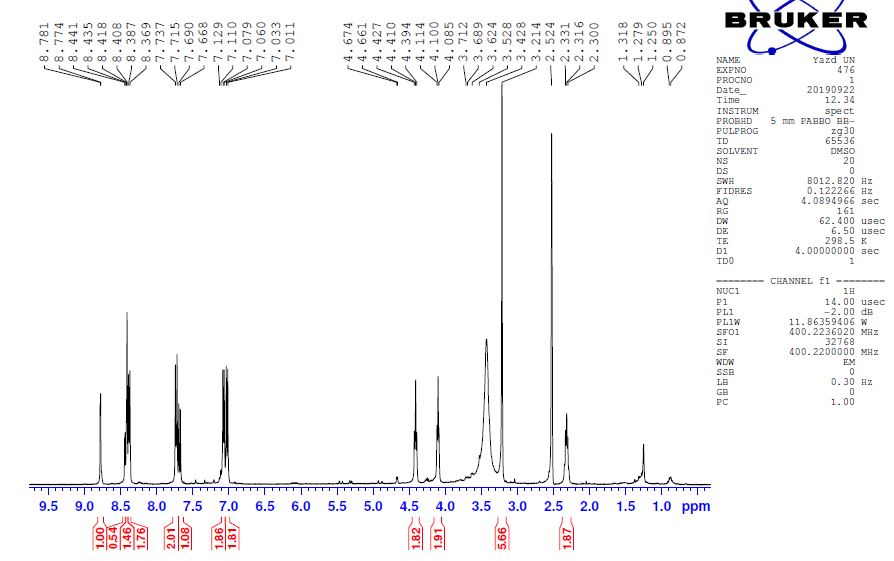
**

**(3t)**

The ^1^H NMR (400 MHz) spectrum of product (3t)


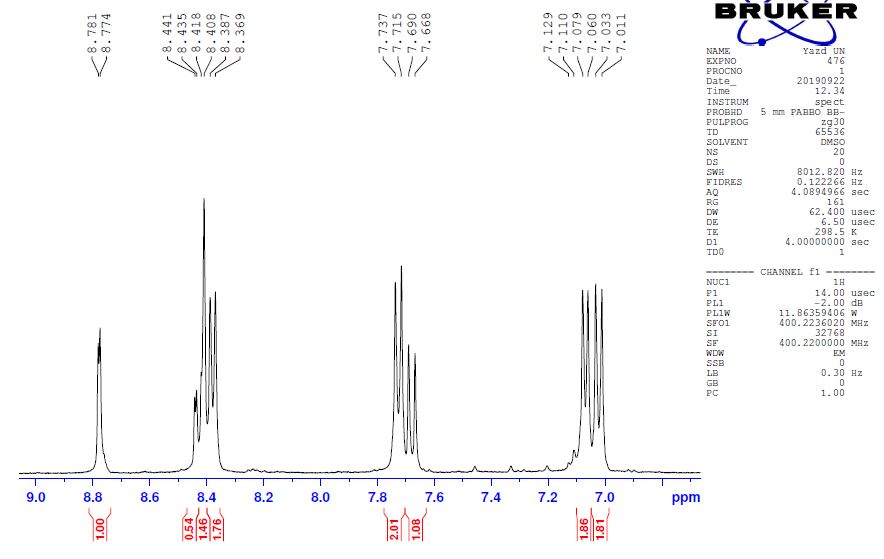


**(3t)**

The ^1^H NMR (400 MHz) spectrum of product (3t)

**
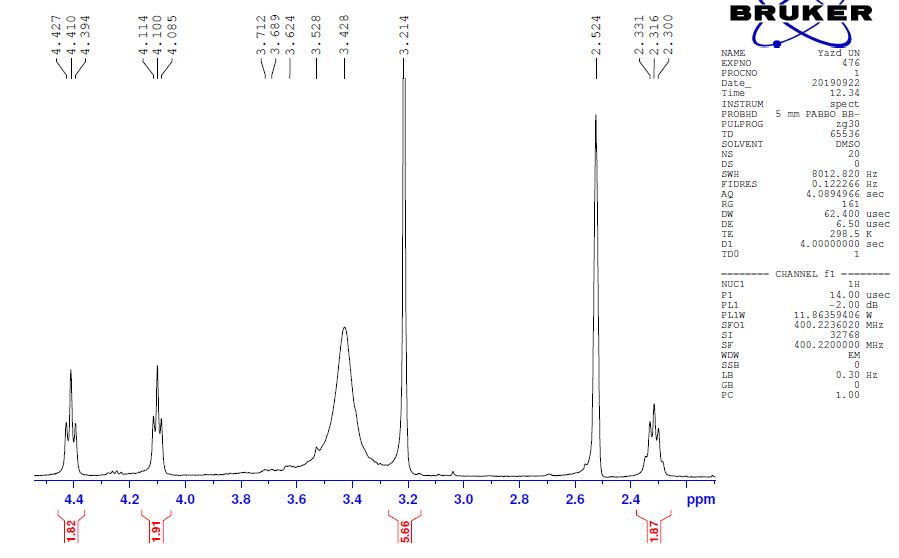
**

**(3t)**

The ^1^H NMR (400 MHz) spectrum of product (3t)


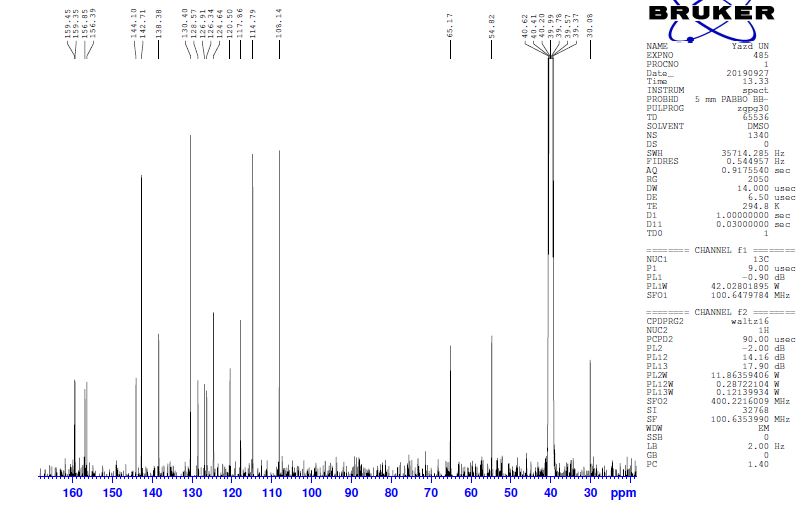


**(3t)**

The ^13^C NMR (100 MHz) spectrum of product (3t)
